# Organelle-Specific Thiochromenocarbazole Imide Derivative as a Heavy-Atom-Free Type I Photosensitizer for Biomolecule-Triggered Image-Guided Photodynamic Therapy

**DOI:** 10.1021/acs.jpclett.5c00136

**Published:** 2025-02-24

**Authors:** Karolina Saczuk, Ahmad Kassem, Marta Dudek, Darío Puchán Sánchez, Lhoussain Khrouz, Magali Allain, Gregory C. Welch, Nasim Sabouri, Cyrille Monnereau, Pierre Josse, Clément Cabanetos, Marco Deiana

**Affiliations:** † Institute of Advanced Materials, Faculty of Chemistry, Wrocław University of Science and Technology, Wyb. Wyspiańskiego 27, 50-370 Wrocław, Poland; ‡ CNRS, MOLTECH-ANJOU, SFR-MATRIX, F-49000 Angers, France; § ENS de Lyon, CNRS, Laboratoire de Chimie, UMR 5182, 46 allée d’Italie, F-69342 Lyon, France; ∥ Department of Chemistry, University of Calgary, 731 Campus Place NW, Calgary, Alberta T2N 1N4, Canada; ⊥ Department of Medical Biochemistry and Biophysics, Umeå University, 90187 Umeå, Sweden; ∇ Department of Medical Biochemistry and Biophysics, Science for Life Laboratory, Umeå University, 90187 Umeå, Sweden

## Abstract

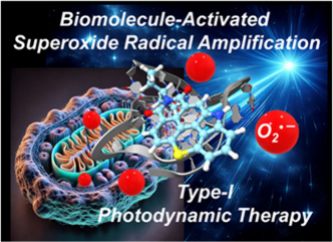

Modern photodynamic therapy (PDT) demands next-generation photosensitizers (PSs) that overcome heavy-atom dependency and enhance efficacy beyond traditional, highly oxygen-dependent type II mechanisms. We introduce herein **TCI-NH**, as a thiochromenocarbazole imide derivative designed for type I photodynamic action. Upon light activation, **TCI-NH** efficiently favors superoxide (O_2_^•–^) and PS-centered radical formation instead of singlet oxygen (^1^O_2_) generation. Its high luminescence efficiency and selective localization in both the endoplasmic reticulum and mitochondria enable precise, image-guided PDT. Notably, interactions with biomolecules, such as serum albumin or DNA, enhance **TCI-NH**’s emission by up to 40-fold and amplify radical generation by up to 5-fold. With negligible dark toxicity, this results in ∼120 nM photocytotoxicity along with an impressive phototherapeutic index exceeding 200. Real-time live-cell imaging revealed rapid, light-triggered cytotoxicity characterized by apoptotic body formation and extensive cellular damage. With its small size, heavy-atom-free structure, exceptional, organelle specificity, and therapeutic efficacy, **TCI-NH** sets a new benchmark for anticancer type I PDT.

Photodynamic therapy (PDT) has emerged as a minimally invasive approach to selectively eradicate cancer cells while largely preserving healthy tissues.^1,2^ Its precision arises from two key principles: targeting light exclusively to the tumor area and utilizing a photosensitizer (PS) that preferentially accumulates in critical organelles of malignant cells.^1,2^ Once illuminated, the PS generates cytotoxic reactive oxygen species (ROS), restricting cellular damage to the desired region and enhancing therapeutic outcomes.^1,2^

PDT operates via two primary mechanisms named type I and type II. Traditionally, PDT has hinged on type II mechanisms, wherein excited PS molecules transfer energy directly to molecular oxygen (O_2_), producing singlet oxygen (^1^O_2_).^3,4^ Early efforts to boost ^1^O_2_ generation heavily relied on the use of large conjugated molecules (porphyrinoids) or the introduction of heavy atoms—halogens or transition metals—into the chromophore, thereby enhancing intersystem crossing (ISC) through spin–orbit coupling (SOC).^2,5^ Yet, while these modifications improved ROS formation, they also increased in dark toxicity, hindered metabolic clearance, involved complex synthesis processes, exhibited limited triplet lifetimes, and often compromised fluorescence.^6−10^ Collectively these drawbacks therefore limited their practical relevance for next-generation PDT.

In response, researchers have shifted their attention toward small organic and heavy-atom-free molecular systems developing inventive strategies to access triplet states without the conventional drawbacks.^6,10^ These approaches include utilizing spin–orbit charge-transfer intersystem crossing (SOCT-ISC) in orthogonal donor–acceptor (D–A) systems,^11−13^ exploiting excitonic coupling to induce excited-state degeneracy and enhance the mixing of singlet and triplet wave functions,^14,15^ employing sulfur substitution (thionation) to tap into nonbonding orbitals,^16−18^ or inducing torsional distortions (twisting-induced ISC) in π-conjugated frameworks.^19−21^ A broad spectrum of structures—from anthracene^22^ and mono- (BTI)^16^ or dibenzothioxanthene imides (DBI)^23,24^ to BODIPY,^22,25^ coumarin,^26^ cyanine,^27^ imidazolium,^28^ naphthalimide,^18^ phenoxazine,^29^ and perylene^30^ derivatives—has emerged. Nonetheless, many of these designs aimed to optimize ^1^O_2_ photogeneration at the expense of fluorescence, which is crucial for real-time imaging and monitoring.^16,18,30^

Within this evolving landscape, crafting a next-generation PS demands a delicate balance: a heavy-atom-free platform that combines robust fluorescence, targeted organelle localization, and efficient ROS generation.^31^ To address this challenge, approaches based on type I mechanisms—which rely on electron transfer to generate radical species such as superoxide (O_2_^•–^), hydroxyl (OH^•^), or hydroperoxyl (HOO^•^)—appear to be advantageous alternatives.^32−34^ Type I PSs typically offer broader therapeutic window and enhanced adaptability across various conditions, including those with limited oxygen availability.^35^ In this context, various design strategies have been employed to develop type I PSs.^35^ For instance, isolated^36−38^ and aggregation-induced emission (AIE)^39−41^ luminogens—with either uncharged or positively charged forms—as well as supramolecular self-assembled dyes^42,43^ responsive to guest interactions^44^ have been synthesized and characterized for type I photodynamic activity. However, some of these systems exhibit hybrid photodynamic mechanisms,^36,38^ often combining type I and type II pathways or incorporating additional effects such as photothermal activation.^37^ This mechanistic ambiguity complicates the interpretation of their photocytotoxicity, making it difficult to ascertain the exclusive contribution of the type I mechanism.

Moreover, several of these type I PSs possess extended chemical structures and high molecular weights,^45,46^ which diverge from the drug-like properties essential for clinical translation. Another concern is that some of these agents exhibit half-maximal inhibitory concentration (IC_50_) values under light irradiation in the 0.5–20 μM range, and some also show significant toxicity in the absence of light.^36,39,40^ These factors collectively constrain their phototherapeutic indices and limit their overall clinical potential.

Building on our previous studies on BTI-^16^ and DBI-based^23,24^ compounds (Figure 1, top panel), which demonstrated outstanding potential for Type I and Type II mechanisms, respectively, we introduce **TCI-NH**, a PS derived from the *N*-annulation process of BTI that exemplifies this new paradigm.^47,48^ Avoiding heavy-atom strategies, **TCI-NH** minimizes ^1^O_2_ formation while excelling at generating O_2_^•–^ and PS-centered radicals through electron transfer/H-abstraction upon light irradiation. Moreover, it selectively localizes within the endoplasmic reticulum (ER) and mitochondria—key organelles responsible for maintaining cellular homeostasis and orchestrating apoptosis.^31^ Notably, when **TCI-NH** coordinates with bovine serum albumin (BSA) or noncanonical DNA structures such as G-quadruplexes (G4s),^49^ both its fluorescence emission and O_2_^•–^ generation are significantly enhanced. This synergy results in pronounced imaging capabilities, potent photocytotoxicity at nanomolar concentrations, negligible dark toxicity, and an outstanding phototherapeutic index. Furthermore, live-cell imaging reveal its rapid induction of cellular damage and apoptotic body formation, highlighting **TCI-NH**’s potential as a powerful, type I PS that integrates bright fluorescence, precise targeting, and robust radical generation. Taken together, these findings illuminate a promising new direction in PDT—one that transcends traditional limitations and embraces safer, more adaptable, and more efficacious treatments.

**Figure 1:**
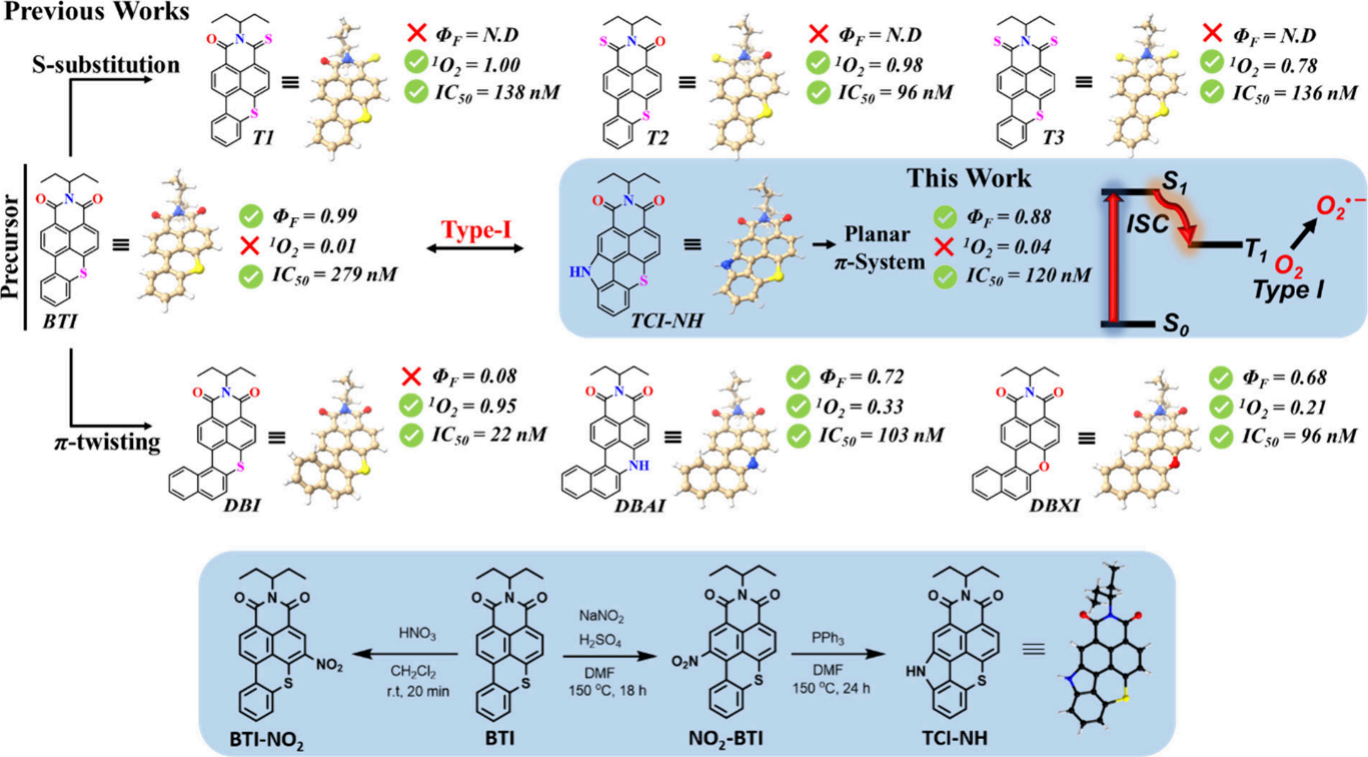
Summary of the BTI-^16^ and DBI-derived^23,24^ structures introduced in previous reports and in the present work, along with their main photophysical features and therapeutic efficacy (top panel). IC_50_ values are derived from experiments conducted on HeLa cells. Synthetic route for the preparation of the target PS **TCI-NH** and the associated crystal structure (bottom panel).

**TCI-NH**, the simplest *N*-annulated BTI derivative featuring a fused, unsubstituted amine,^47,48^ was synthesized by revisiting the previously reported nitration conditions^50^ used to obtain BTI-NO_2_ (Figures S1–S6). These modified conditions enabled the direct and selective introduction of a nitro group in the bay position without the need for prior protection. As outlined in Figure 1 (bottom panel), the expected NO_2_–BTI was successfully isolated by replacing nitric acid with a mixture of sodium nitrite and sulfuric acid. A subsequent Cadogan cyclization afforded the target compound, which was crystallized by slow evaporation. X-ray diffraction then confirmed the structure of the resulting **TCI-NH** (Figure 1 and Supplementary Table 1).

The absorption and emission spectra, along with the photophysical properties of **TCI-NH** (excluding those involving biological templates or cellular studies, *vide infra*), were recorded in chloroform (CHCl_3_)—a solvent of moderate polarity that effectively solubilizes the compound while minimizing aggregation effects (Figure 2). Compared to previously reported data for BTI under similar conditions,^16,50^**TCI-NH** exhibited a bathochromic shift in its absorption maximum, observed at 480 nm compared to 455 nm for BTI (Table 1). In contrast, the emission maximum remained essentially unchanged at 510 nm for both BTI and **TCI-NH**.

**Figure 2:**
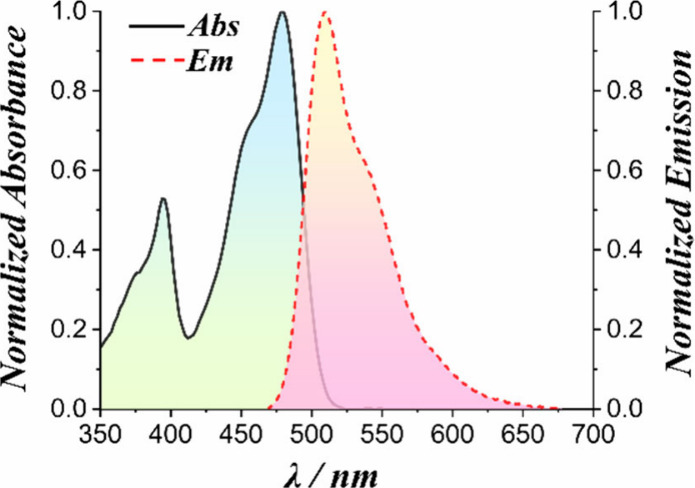
UV–vis absorption and emission spectra of **TCI-NH** in CHCl_3_.

**Table 1:** Photophysical, Biophysical, and Cell-Related Parameters of BTI and **TCI-NH**

Cmp	λ_max_/nm	λ_em_/nm	τ/ns	*Φ*_*F*_^a^	*Φ*_*Δ*_^b^	IC_50_/nM^c^
BTI	455	510	7.5	0.99	0.01	279.0
TCI-NH	480	510	7.3	0.88	0.04	120.8

a *Φ*_*F*_ = measured using Coumarin-153 as reference (*Φ*_*F*_ = 0.45 in MeOH).

b *Φ*_*Δ*_ = Measured using Phenalenone as reference (*Φ*_*Δ*_ = 0.95 in CHCl_3_).

c IC_50_ was determined in HeLa cells with excitation wavelength of 470 ± 22 nm and operating at an intensity of 27 mW/cm^2^ for 6 min.

Whereas BTI displays a fluorescence quantum yield, *Φ*_*F*_, approaching unity ∼0.99, **TCI-NH** showed a slightly reduced value ∼0.88 (Figure S7). This decrease is likely attributable to an increased contribution of ISC to the excited-state deactivation pathway, as reflected by an improvement in singlet oxygen quantum yield, *Φ*_*Δ*_ (*vide infra*). Nonetheless, similar to BTI, **TCI-NH** exhibited a relatively long excited-state lifetime of 7.3 ns.

The outstanding emission properties of **TCI-NH** prompted us to investigate its intracellular distribution in live human cervical carcinoma (HeLa) cells using confocal laser scanning microscopy (CLSM) (Figure 3). For biological experiments, **TCI-NH** was dissolved in DMSO to prepare a stock solution—leveraging DMSO’s ability to solubilize both hydrophobic and hydrophilic compounds, its miscibility with water and cell culture medium, and its low cytotoxicity in HeLa cells at concentrations ≤0.5% v/v.^16,23,24^ Cells were treated with 500 nM **TCI-NH**, incubated for 24 h, and washed with phosphate-buffered saline (PBS) to remove unbound **TCI-NH** molecules. This incubation period was selected based on phototoxicity studies (*vide infra*) to precisely match the delay before illumination for photoinduced cell death, ensuring reproducible accumulation and localization patterns prior to irradiation.

**Figure 3:**
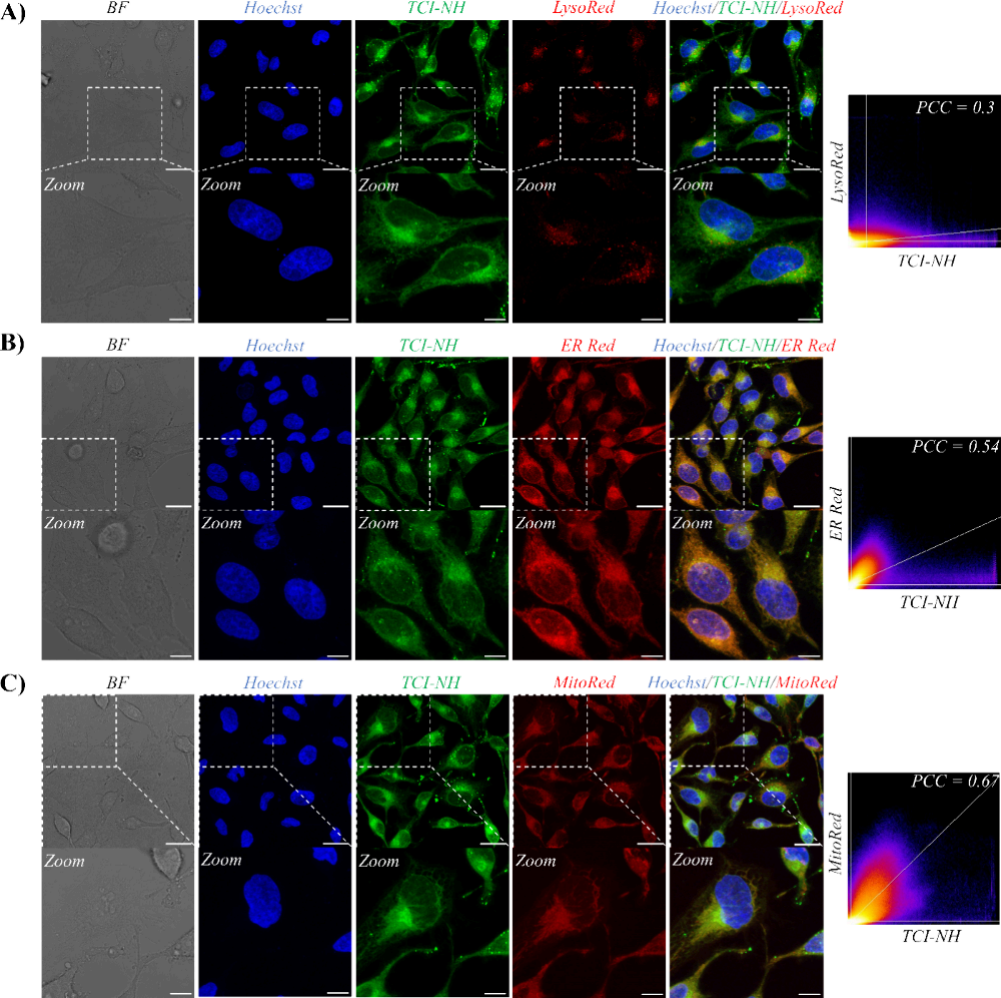
Cellular localization of **TCI-NH** in HeLa cells. **A-C)** CLSM images showing the intracellular distribution of **TCI-NH** (500 nM, green fluorescence) in live HeLa cells after a 24-h incubation. Following treatment, cells were costained with the nuclear dye Hoechst 33342 (500 nM, blue fluorescence) and one of the following organelle-specific dyes (red fluorescence): Lyso-Tracker Red (100 nM, panel A), ER-Tracker Red (500 nM, panel B), or Mito-Tracker Red (100 nM, panel C). Scatter plots illustrate the quantification of colocalization between **TCI-NH** and each organelle stain, calculated using the PCC. Excitation and emission settings for fluorescence detection were 405/420–470 nm for Hoechst 33342, 480/495–560 nm for **TCI-NH**, 577/600–710 nm for Lyso-Tracker Red, 587/600–710 nm for ER-Tracker Red, and 580/590–715 nm for Mito-Tracker Red. Scale bars are 25 μm for main images and 10 μm for magnified views.

Under these conditions, **TCI-NH** primarily localized in the cytoplasm, where it formed discrete, highly emissive species (Figure 3, green channel). To gain deeper insight into its organelle-specific distribution, live HeLa cells were costained with four commercially available organelle-selective probes namely Hoechst 33342 (nuclei), ER-Tracker Red (ER), Lyso-Tracker Red (lysosomes), and Mito-Tracker Red (mitochondria). While negligible fluorescence overlap between **TCI-NH** and Hoechst 33342 confirmed a minimal nuclear uptake, the low Pearson’s correlation coefficient (PCC) with Lyso-Tracker Red indicated limited lysosomal localization (Figure 3A).

In contrast, and as illustrated in Figure 3B–C, the green emission of **TCI-NH** displayed notable overlap with both ER-Tracker Red and Mito-Tracker Red signals, resulting in PCC values of 0.54 and 0.67, respectively. These data indicate that **TCI-NH** effectively accumulates in both the ER and mitochondria. Besides, **TCI-NH** exhibited intense, punctate fluorescent clusters in the cytoplasm that did not align with any of the tested organelle trackers. Such signals may correspond to lipid-rich vesicular structures, including multivesicular bodies (MVBs)^51^ and/or lipid droplets (LDs).^52^ Their presence somewhat reduced the measured PCC values since these compartments are not associated with either the ER or mitochondria.

Building on the pronounced fluorescence emission of **TCI-NH** in both the ER and mitochondria, we next investigated how its interactions with cellular biomolecules might influence its subcellular localization. These organelles are not only enriched in proteins but, in the case of mitochondria, also contain their own genetic material (mtDNA), which is notably guanine-rich and to forming G4 structures^53−55^—motifs that have attracted considerable attention as potential anticancer targets,^49,56^ and have been shown our previous work to strongly interact with other BTI derivatives.^23^ To probe the potential influence of these biomolecules, we performed fluorescence titration binding experiments using BSA as a model protein,^18,33^ along with three major types of DNA structures, double-stranded DNA (dsDNA), single-stranded DNA (ssDNA), and G4 DNA (Figure 4).

**Figure 4:**
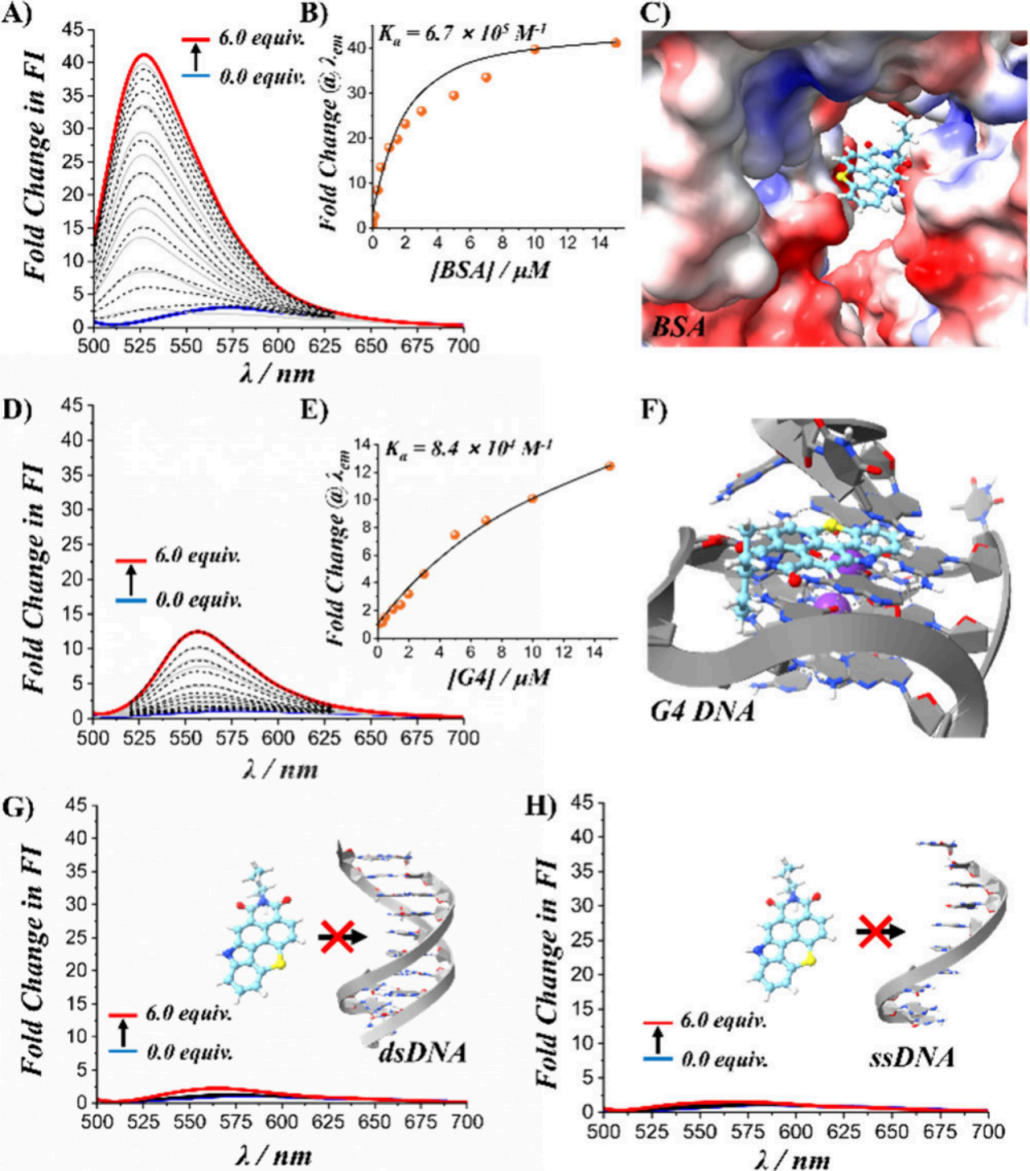
Fluorescence titration binding studies. **A-C)** Fluorescence spectral changes of **TCI-NH** (2.5 μM) with increasing BSA concentrations (0 to 15 μM), accompanied by the corresponding 2:1 binding isotherm and a schematic illustration depicting **TCI-NH** accommodated within the hydrophobic pocket of BSA (PDB: 4OR0). **D-F)** Fluorescence spectral changes of **TCI-NH** (2.5 μM) with increasing G4 DNA concentrations (0 to 15 μM), along with the associated 1:1 binding isotherm and a schematic representation showing **TCI-NH** binding to the external tetrad end of the G4 structure (PDB: 1XAV). **G-H)** Fluorescence titration studies demonstrating that **TCI-NH** does not form complexes with dsDNA (G) or ssDNA (H). All the experiments were carried out in 50 mM Tris-phosphate buffer (pH 6.8) supplemented with 100 mM KCl at 25 °C.

In a buffered aqueous solution, **TCI-NH** exhibited only a weak emission with a maximum at approximately 580 nm, strongly red-shifted compared to data in CHCl_3_ solutions (510 nm), likely due to the formation of molecular aggregates.^16,18,57^ Addition of BSA led a pronounced ∼40-fold fluorescence enhancement and a ∼50 nm blue shift, resulting in a **TCI-NH**–BSA complex emitting around 527 nm (Figure 4A-C). This strong fluorescence increase, associated with the hypsochromic shift, suggests that **TCI-NH** becomes disassembled^16,18,57^ and is subsequently encapsulated within a hydrophobic protein pocket, thereby effectively shielding it from the aqueous environment. Such sequestration likely reduces polarity and restricts rotational freedom, thus stabilizing the excited state and thereby enhancing emission. Similar binding modes have been documented in the literature. For example, Liu and co-workers reported that a 2′-hydroxychalcone derivative initially exhibited aggregation-caused quenching (ACQ) with a red-shifted emission band and underwent disassembly upon binding to human serum albumin (HSA).^58^ Subsequently, the disassembled probe molecules were encapsulated within the hydrophobic cavity of HSA, which led to a marked fluorescence enhancement and a blue shift. The close agreement between these observations and our findings provided a strong basis for our mechanistic interpretation.

A more moderate response was observed upon titration with G4 DNA. Under these conditions, the fluorescence intensity increased by about 12-fold and the emission maximum blue-shifted ∼20 nm, to approximately 557 nm (Figure 4D-F). The reduced magnitude of the spectral shifts, compared to the BSA complex, suggests that although **TCI-NH** can engage in π-stacking interactions with the terminal guanine tetrads—as observed for its closely related analogue DBI^23^ and numerous other ligands^59−62^—it remains partially exposed to the aqueous environment. In contrast, no significant interaction occurred with dsDNA or ssDNA, likely due to the absence on **TCI-NH** of necessary positively charged moieties for strong electrostatic attraction with the DNA backbone (Figure 4G-H).

Global nonlinear curve-fitting analyses of the titration data yielded association constants (*K*_*a*_) of about 6.7 × 10^6^ M^–1^ for **TCI-NH**–BSA and 8.4 × 10^4^ M^–1^ for **TCI-NH**–G4 complexes (Figure 4B and E), reinforcing the physiological relevance of these interactions.

After establishing the photophysical properties, subcellular localization, and potential biomolecular interactions of **TCI-NH**, we next assessed its capacity to act as a light-activated theranostic agent in anticancer research. We began by evaluating its ability to produce ROS in test tube settings with an initial focus on ^1^O_2_ generation, which has been a well-documented mechanism in BTI- and DBI-based compounds.^16,23,24^

To directly monitor ^1^O_2_ production, we measured the characteristic phosphorescence emission at ∼1270 nm in CHCl_3_ (Figure S8). Under these conditions, **TCI-NH** exhibited a modest *Φ*_*Δ*_ of about 4%, consistent with the negligible ^1^O_2_ generation previously observed for its BTI precursor.^16^ To further verify this result in aqueous media, we employed 9,10-anthracenediyl-bis(methylene)dimalonic acid (ABDA) as a specific ^1^O_2_ sensor since it can be readily oxidized and bleached by ^1^O_2_, resulting in a decrease in its absorption that can be easily tracked by UV/vis spectroscopy (Figure S9).^63^

In the presence of **TCI-NH**, ABDA bleaching was minimal, providing a *Φ*_*Δ*_ of about 7% corroborating the direct ^1^O_2_ measurements and confirming that **TCI-NH** is a poor ^1^O_2_ PS. We also examined whether complexation with biological macromolecules could enhance ^1^O_2_ generation, as **TCI-NH** is likely to be protein- or DNA-bound in the cellular environment.^57^ To this end, we tested **TCI-NH** complexed with BSA and with G4 DNA and it appeared that neither the **TCI-NH**–BSA nor the **TCI-NH**–G4 complex induced significant ABDA bleaching, remaining in line with the low *Φ*_*Δ*_ (Figure S9). These findings suggested that, while **TCI-NH** interacts effectively with biological targets, this interaction does not substantially improve its ^1^O_2_ generation ability, making it a poor candidate for Type II PDT.

Prompted by the limited ^1^O_2_ production, we turned to explore alternative ROS generation pathways, particularly O_2_^•–^, which is often generated via a Type I mechanism. To investigate O_2_^•–^ generation, we employed Dihydrorhodamine-123 (DHR-123), a nonfluorescent probe that becomes highly fluorescent upon oxidation to rhodamine-123 by O_2_^•–^.^33^ Under light irradiation, **TCI-NH** induced a gradual enhancement in DHR-123 fluorescence, reaching ∼20-fold after 300 s (Figure 5A). This clear fluorescence upturn confirmed that **TCI-NH** can efficiently produce O_2_^•–^ radicals.

**Figure 5:**
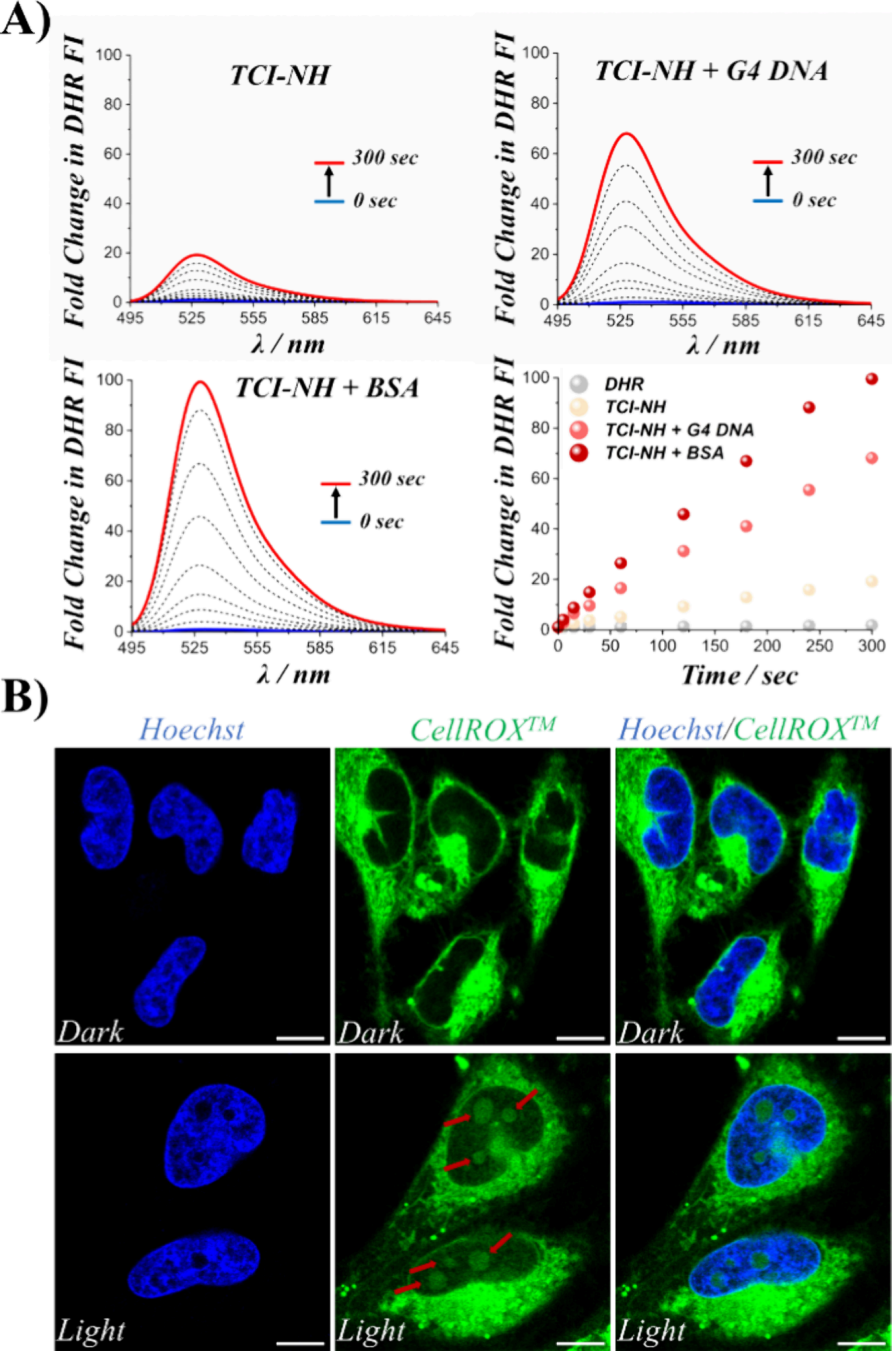
**A)** Detection of O_2_^•–^ using DHR-123 as a sensor. Turn-on fluorescence activation of DHR-123 (5 μM) was monitored as an indicator of O_2_^•–^ generation by **TCI-NH** (5 μM) upon irradiation. Samples were irradiated at various time intervals (0–300 s) using excitation at 485 ± 20 nm. **TCI-NH** experiments were conducted in the presence of BSA (10 μM) or G4 DNA (10 μM) in Tris-HCl buffer (50 mM, pH 6.8) with 100 mM KCl. **B)** Intracellular ROS generation induced by **TCI-NH**. HeLa cells were treated with **TCI-NH** (0.5 μM) and CellROX Green reagent (5 μM). Where specified, blue light irradiation (6 min) was applied using a LED light cube and cells were fixed with 4% paraformaldehyde (PFA). Fluorescence imaging was performed with excitation/emission wavelengths of 490/510–650 nm. Note that the extranuclear green fluorescence observed in **TCI-NH**-treated cells corresponds to the intrinsic fluorescence of **TCI-NH**, which coincides with that of CellROX. In contrast, the nuclear and nucleolar green fluorescence, highlighted by red arrows, indicates **TCI-NH** photoinduced oxidation of the CellROX Green reagent. Scale bar: 10 μm.

Next, we tested whether the **TCI-NH**–BSA or **TCI-NH**–G4 complexes could further enhance O_2_^•–^ generation (Figure 5A). Remarkably, the **TCI-NH**–G4 complex boosted the DHR-123 fluorescence by about 70-fold after 300 s, representing a 3.5-fold increase compared to unbound **TCI-NH**. Even more strikingly, the **TCI-NH**–BSA complex elicited almost a 100-fold rise in DHR-123 oxidation, a 5-fold improvement over the free **TCI-NH**. These data strongly indicate that biomolecular interactions not only guide **TCI-NH** localization and enhance emission but also significantly amplify its capacity to photogenerate O_2_^•–^ radicals.^33,57^ Such enhancement aligns with existing literature reports for other PSs, where protein or DNA binding modifies the photophysical properties and ROS output.^18,33,57^

Finally, to confirm that **TCI-NH**’s O_2_^•–^-generating ability observed in test tubes translates into cellular contexts, we employed CellROX—a nonfluorescent, green-emitting dye that becomes fluorescent upon oxidation by ROS and then localizes within the nucleus due to DNA binding.^16,23,24^ In the absence of light, cells incubated with **TCI-NH** and CellROX displayed no nuclear fluorescence, in line with a lack of photo-oxidative damage (Figure 5B). However, upon irradiation, cells treated with **TCI-NH** and CellROX showed strong nuclear fluorescence, confirming that **TCI-NH** is capable of photoinducing ROS within live cells.

The ability of **TCI-NH** to generate O_2_^•–^, combined with its preferential localization within the ER and mitochondria, led us to evaluate its potential photocytotoxic effects and more precisely, its capacity to selectively induce cell death under light irradiation, following a Type I PDT mechanism. Both the ER and mitochondria are pivotal in determining cellular fate. Mitochondria maintain membrane potential, modulate metabolic activity, and help govern apoptotic pathways through the delicate balance of ROS levels.^31,64,65^ In parallel, the ER regulates protein and lipid biosynthesis, calcium homeostasis, and the cellular stress response.^31,66,67^ Photoinduced oxidative stress in the ER can release stored calcium, triggering apoptotic signaling, while mitochondrial damage often leads to the release of pro-apoptotic factors and a surge in ROS.^31^ These organelles are functionally interconnected at mitochondria-associated membranes (MAMs), ensuring that damage to one can influence the other, thereby orchestrating a potent, integrated cell death response.^68^

To evaluate **TCI-NH**’s phototherapeutic effectiveness, we conducted cytotoxicity experiments in HeLa cells (Figure 6). Under dark conditions, incubations with **TCI-NH** at concentrations up to 25 μM for 48 h produced no apparent toxicity, and cell viability was essentially unaffected. In stark contrast, when cells were treated with significantly lower **TCI-NH** concentrations (0.0121 μM to 1.56 μM) for 24 h and subsequently exposed to light, viability decreased dramatically, yielding an IC_50_ of 120.8 ± 8.6 nM. Notably, the timing of irradiation (after 24 h) coincided with the period during which **TCI-NH** was confirmed to be colocalized in the ER and mitochondria, thereby ensuring that the observed cell death could be attributed to targeted photodynamic action at these organelles level. Overall, these results highlight a phototherapeutic index of at least 200-fold, underscoring **TCI-NH**’s significant potential as an anticancer PS operating predominantly via a Type I (O_2_^•–^-mediated) mechanism.

**Figure 6:**
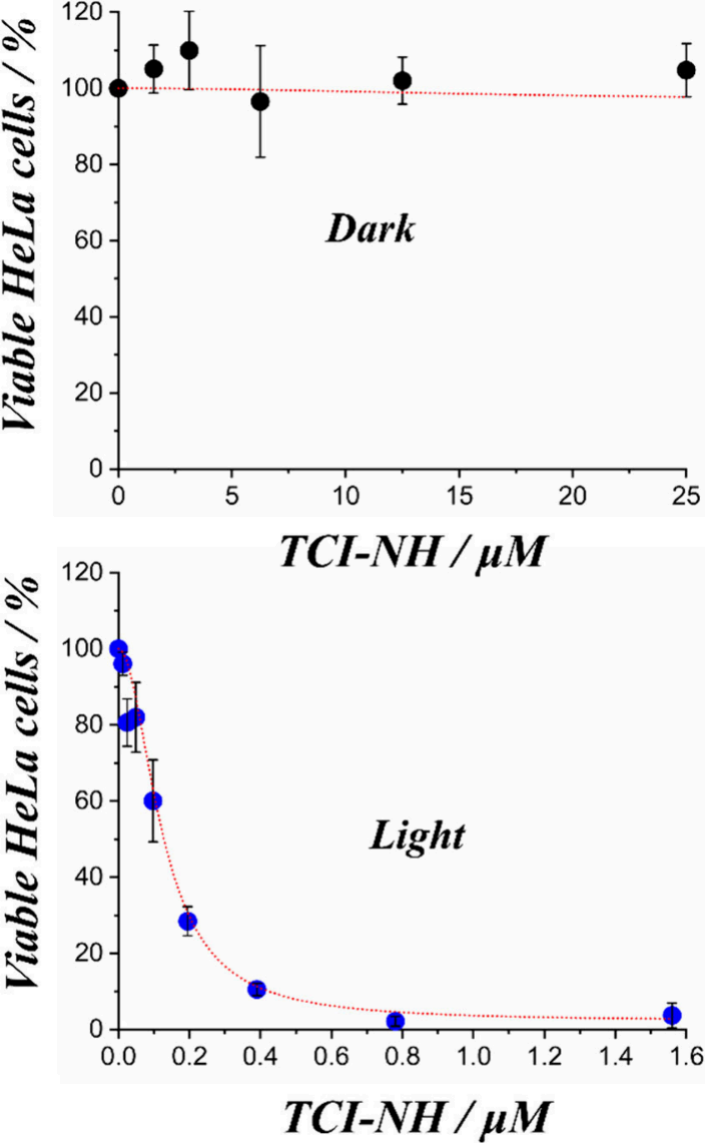
(Photo)toxicity studies in HeLa cells. Cell viability was assessed in HeLa cells treated with varying concentrations of **TCI-NH**, either in the absence (dark conditions) or presence (light conditions) of blue light irradiation for 6 min. The results are expressed as mean ± standard deviation (*n* = 3), with error bars indicating variability across replicates.

To gain insight into the mode of cell death triggered by **TCI-NH**, we performed real-time morphological assessments of treated cells (Figure S10). Under dark conditions, **TCI-NH**-treated cells remained morphologically indistinguishable from controls, consistent with its negligible dark toxicity. However, under continuous irradiation of the same area, cells began to exhibit hallmarks of apoptotic cell death.^9,16,23,24^ Distinct morphological alterations were indeed evident, including membrane blebbing and the formation of apoptotic bodies.^69^ These changes are classic indicators of apoptosis and strongly support the notion that **TCI-NH**-induced ROS generation, when activated by light, can drive an apoptotic response, which aligns well with previously reported analogues.^16,23,24^

Control experiments using DMSO-treated cells under identical illumination conditions showed no significant morphological alterations, confirming that the observed changes were due to the light-dependent activity of **TCI-NH** (Figure S10).

To further elucidate the mechanisms contributing to the robust PDT activity of **TCI-NH**—despite its relatively low ^1^O_2_ generation—we conducted electron paramagnetic resonance (EPR) experiments in CHCl_3_ and DMSO. This approach was motivated by previous studies, which demonstrated that variations in solvent polarity, can significantly influence the photogeneration of distinct ROS.^70^ 2,2,6,6-tetramethylpiperidine (TEMP) was employed as a ^1^O_2_ scavenger in CHCl_3_, with the increase in the characteristic 2,2,6,6-tetramethylpiperidin-1-oxyl (TEMPO) radical signature monitored to quantify ^1^O_2_ generation efficiency.^16,23,24^ In parallel, 5,5-dimethyl-1-pyrroline N-oxide (DMPO) was primarily used to characterize the formation of O_2_^•–^ and related radical oxygen species (HO^•^, HOO^•^) in DMSO.^16,23,24^ In all cases, experiments were conducted on **TCI-NH** as well as DBI and BTI, which were used as benchmark photosensitizers for ^1^O_2_^23^ or O_2_^•–^^16^ production, respectively.

As a result, EPR spectra upon constant irradiations of each tested PS in the presence of TEMP clearly confirmed our initial findings: while DBI exhibits very high efficient ^1^O_2_ generation efficiency,^23^**TCI-NH** show a, comparable to that of BTI (Figure S11).

In striking contrast, no significant buildup of DMPO-adduct signals was observed for DBI, whereas both BTI and **TCI-NH** demonstrated effective radical production, albeit with notable differences (Figure 7). The DMPO-adducts formed during BTI irradiation (Figure 7A and Figure S12A) can be attributed to a mixture of O_2_^•–^ (g = 2.006, aN(G)=13.45, aH = 8.25, aH = 1.6, 55%) and HOO^•^ (g = 2.006, aN(G)=13.3, aH = 9.5, aH = 1, 45%) radicals.^71,72^ In the case of **TCI-NH** (Figure 7B and Figure S12B) a distinct and intense signature of an N-centered radical adduct was additionally observed alongside the O_2_^•–^ and HOO^•^ adducts signal, and characterized by a unique hyperfine splitting pattern (g = 2.006, aN(G)=14.4, aH = 21, 30%) that can reasonably be attributed to the formation of a **TCI-N**^**•**^ radical. Combined with the results of biological experiments, these findings suggest that a Type I mechanism underlies the highly efficient photoinduced cell death observed with **TCI-NH**.

**Figure 7:**
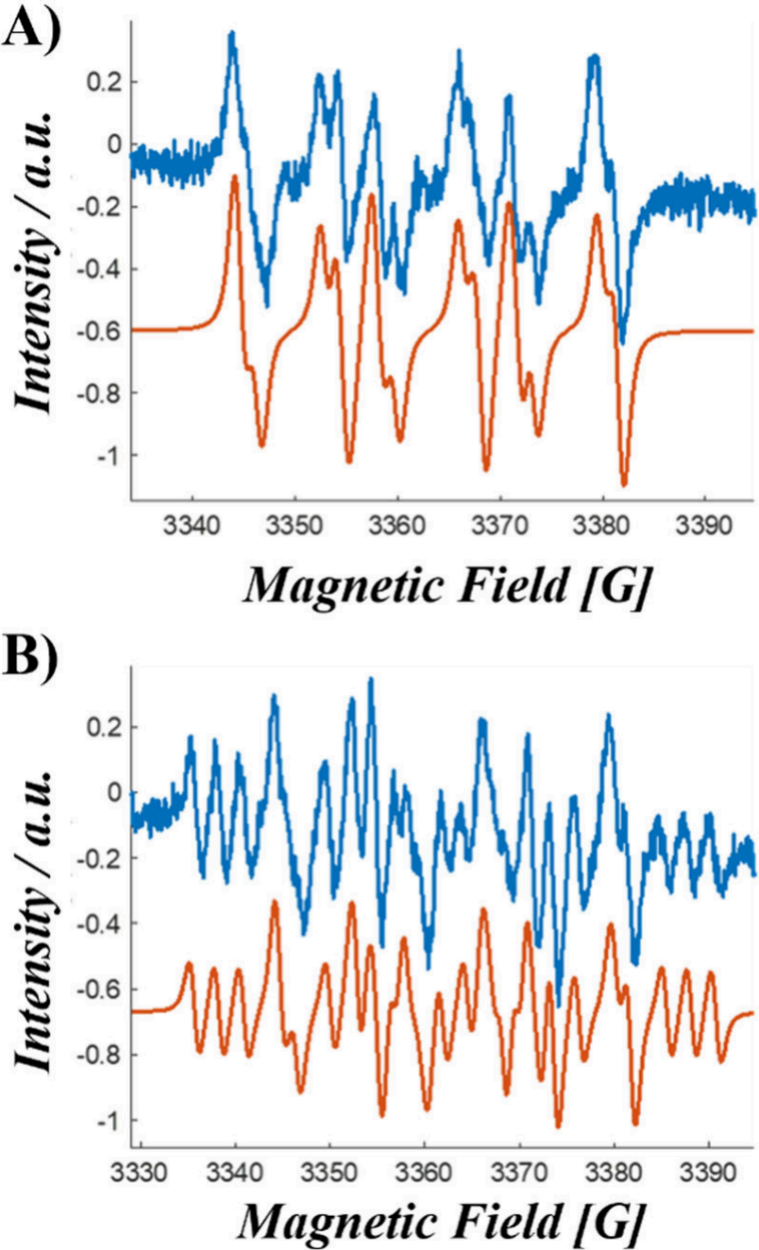
Experimental (blue) and simulated (red) EPR signals of DMPO adducts obtained under 455 nm illumination of DMSO solutions containing **A)** BTI and **B) TCI-NH**, highlighting the photoinduced generation of (A) O_2_^•–^ (g = 2.006, aN(G)=13.45, aH = 8.25, aH = 1.6, 55%) and HOO^•^ (g = 2.006, aN(G)=13.3, aH = 9.5, aH = 1, 45%) and (B) O_2_^•–^ (g = 2.006, aN(G)=13.5, aH = 8.2, aH = 1.65, 40%), HOO^•^ (g = 2.006, aN(G)=13.3, aH = 9.5, aH = 1, 30%) and **TCI-N**^**•**^ (g = 2.006, aN(G)=14.4, aH = 21, 30%).

In conclusion, this work introduces **TCI-NH** as a next-generation, heavy-atom-free PS that exemplifies a strategic pivot away from conventional, predominantly type II photodynamic pathways. Unlike traditional ^1^O_2_-based approaches, **TCI-NH** efficiently generates both O_2_^•–^ and PS-centered radicals under light activation, thereby expanding the available toolkit for ROS-mediated tumor ablation. Its capacity to target both the ER and mitochondria, a tandem of organelles integral to apoptotic signaling, ensures a potent and synergistic mode of cellular assault. The result is rapid and light-driven cell death easily characterized by distinct apoptotic features, including pronounced membrane blebbing, formation of apoptotic bodies, and nuclear deformation.

Crucially, **TCI-NH**’s phototoxicity is amplified by interactions with proteins and G4 DNA domains, highlighting the importance of selective biomolecular binding in refining subcellular localization and increasing ROS yield. The ability of these complexes to enhance fluorescence and O_2_^•–^ generation provides a powerful avenue for both image-guided therapy and improved therapeutic indices. Remarkably, **TCI-NH** achieves high photocytotoxicity with minimal dark toxicity and an impressive phototherapeutic index exceeding 200, an accomplishment that underscores its translational potential.

Collectively, these findings position the **TCI-NH** molecular design as a compelling theranostic strategy for next-generation PSs, seamlessly integrating bright fluorescence imaging with efficient protein- and DNA-assisted radical generation to enable safer, more effective, and versatile treatments against malignant cells. To further advance the therapeutic outcome and accelerate clinical translation, we have initiated studies in more sophisticated *in vivo* models. Our recent investigations using both wild type and transgenic zebrafish harboring rhabdomyosarcoma tumors have yielded very promising phototherapeutic outcomes, with detailed results to be published in the near future.

## Supporting Information Available

Experimental details, synthesis and characterization, crystallographic data, ABDA bleaching experiments, dynamic imaging of cellular morphology changes and EPR data (PDF)
Transparent Peer Review report available (PDF)
