# Supplementary material for: Organelle-Specific Thiochromenocarbazole Imide Derivative as a Heavy-Atom-Free Type I Photosensitizer for Biomolecule-Triggered Image-Guided Photodynamic Therapy
Source: J Phys Chem Lett. 2025 Feb 24;16(9):2273–82. doi: 10.1021/acs.jpclett.5c00136 (PMC11891978; doi:10.1021/acs.jpclett.5c00136)
Supplement: Supplementary file 1 — jz5c00136_si_001.pdf [file jz5c00136_si_001.pdf]

Supporting Information

Organelle-Specific Thiochromenocarbazole Imide Derivative as a Heavy-Atom-Free Type I Photosensitizer for Biomolecule-Triggered Image-Guided Photodynamic Therapy

Karolina Saczuk,[#][a] *Ahmad Kassem,*[#][b] *Marta Dudek,*[a] *Darío Puchán Sánchez,*[b] *Lhoussain Khrouz,*[c] *Magali Allain,*[b] *Gregory C. Welch,*[d] *Nasim Sabouri,*[e] *Cyrille Monnereau,*[c] *Pierre Josse,**[b] *Clément Cabanetos**[b] *and Marco Deiana**[a,e]

[a] Institute of Advanced Materials, Faculty of Chemistry, Wrocław University of Science and Technology, Wyb. Wyspiańskiego 27, 50-370 Wrocław, Poland

[b] CNRS, MOLTECH-ANJOU, SFR-MATRIX, F-49000 Angers, France

[c] ENS de Lyon, CNRS, Laboratoire de Chimie, UMR 5182, 46 allée d'Italie, F-69342 Lyon, France

[d] Department of Chemistry, University of Calgary, 731 Campus Place NW, Calgary, Alberta, Canada

[e] Department of Medical Biochemistry and Biophysics, Umeå University, 90187 Umeå, Sweden

Corresponding Authors

*pierrejosse@yahoo.fr ; *clement.cabanetos@cnrs.fr ; *m.deiana@pwr.edu.pl

[#]These authors contributed equally.

Experimental Details

Thin Layer Chromatographies (TLCs) were performed on pre-coated aluminium sheets with 0.20 mm Merck Alugram SIL G/UV254 under UV @ 254 nm. Column chromatography purifications were carried out using Sigma-Aldrich silica gel 60 (particle size 63-200 µm). Nuclear magnetic resonance (NMR) ^{1}H and ^{13}C spectra were obtained on a Bruker 300 MHz Avance III spectrometer (300 MHz for ^{1}H and 75 MHz for ^{13}C). Chemical shifts were reported in ppm according to tetramethylsilane using the solvent residual signal as an internal reference ($CDCl_3$: δH = 7.26 ppm, δC = 77.16 ppm). Coupling constants (J) were given in Hz. NMR spectra were measured at 298 K. Resonance multiplicity was described as s (singlet), d (doublet), t (triplet), m (multiplet), dd (doublet of doublets), tt (triplet of triplets), td (triplet of doublets), and br (broad signal). Carbon spectra were acquired with a complete decoupling for the proton. High resolution mass spectrometry (HRMS) was performed with a JEOL JMS-700 B/E.

Synthesis and Characterization

The BTI was prepared using our early reported synthetic method.[1]

Synthesis of NO_2-BTI: To a stirred solution of BTI (100 mg, 1 equiv.) and sodium nitrite (184 mg, 10 equiv.) in DMF (5 mL) at 0 °C was added sulfuric acid (143 µL, 10 equiv.) dropwise. The mixture was stirred at 0 °C for 5 minutes and then heated to 150 °C for 18 hours. After cooling down to room temperature, the mixture was diluted with dichloromethane (approx. 30 mL) and transferred into a decantation flask where it was washed with water (2x) and brine (1x). The organic phase was dried over magnesium sulfate and concentrated by rotary evaporation. The crude product was then subjected to silica gel column chromatography, using dichloromethane as eluent, to afford of the two different nitrated regio isomers (56 mg, 50 % yield), corresponding to BTI-NO_2 and NO_2-BTI, and isolated in a 1:1.8 ratio, respectively.

^{1}H NMR (300 MHz, $CDCl_3$): δ 8.89 (s, 1H), 8.53 (d, *J* = 8.0 Hz, 1H), 7.76 (d, *J* = 8.0 Hz, 1H), 7.63 (dd, *J* = 7.9, 1.3 Hz, 1H), 7.58 (dd, *J* = 8.1, 1.5 Hz, 1H), 7.50 (td, *J* = 7.6, 1.3 Hz, 1H), 7.39 (ddd, *J* = 8.5, 7.2, 1.6 Hz, 1H), 5.09 – 4.97 (m, 1H), 2.23 (m, 2H), 1.91 (m, 2H), 0.89 (t, *J* = 7.5 Hz, 6H).

^{13}C NMR (75 MHz, $CDCl_3$): δ 145.9, 140.6, 138.0, 133.6, 132.1, 131.6, 131.1, 130.6, 130.1, 129.8, 129.1, 128.3, 127.9, 127.7, 127.20, 125.4, 125.0, 122.3, 118.8, 58.0, 24.9, 11.4.

HRMS (MALDI-TOF) calculated for $C_{23}H_{18}N_2O_4S$ 418.09917, found 418.09928 (mass accuracy = -0.32 ppm).

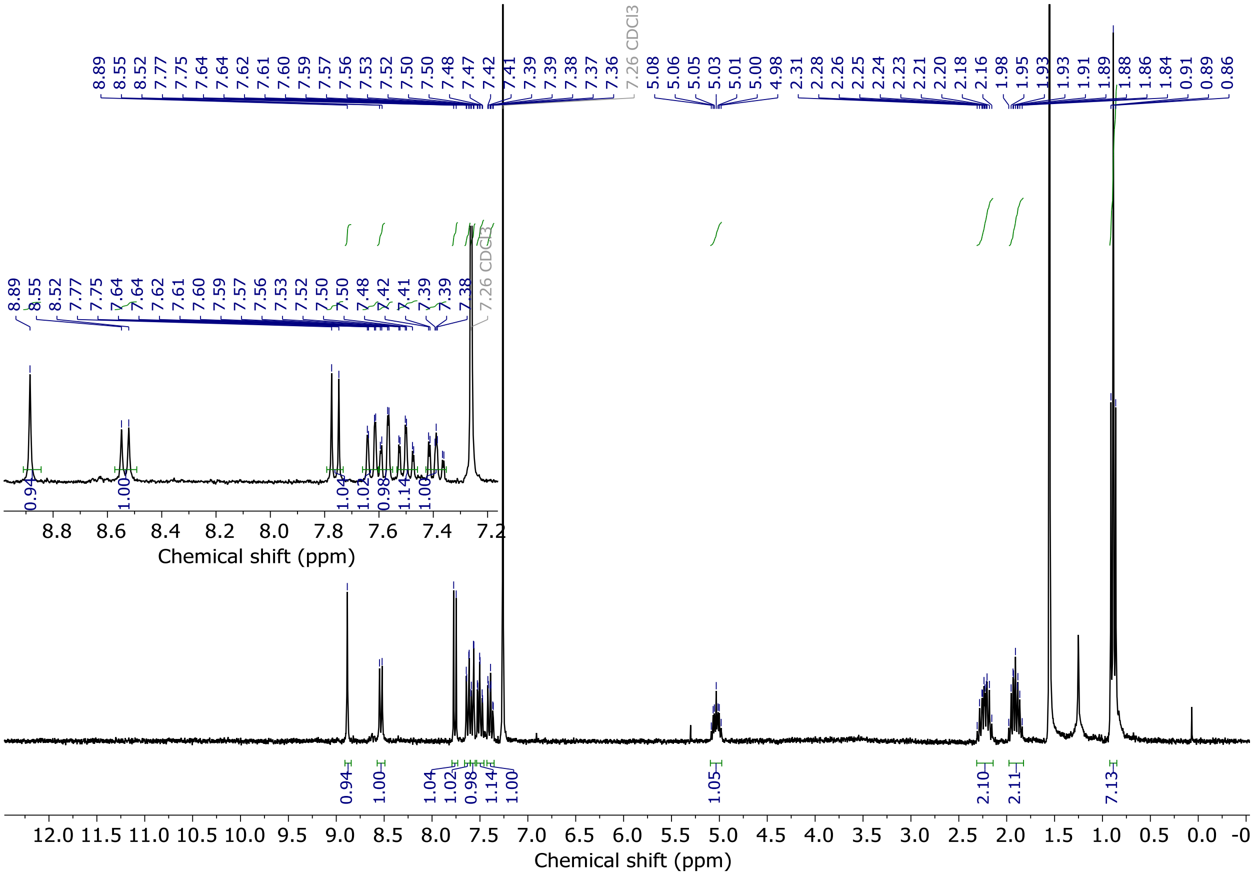

Figure S1. ^{1}H NMR ($CDCl_3$) of **NO_2-BTI**

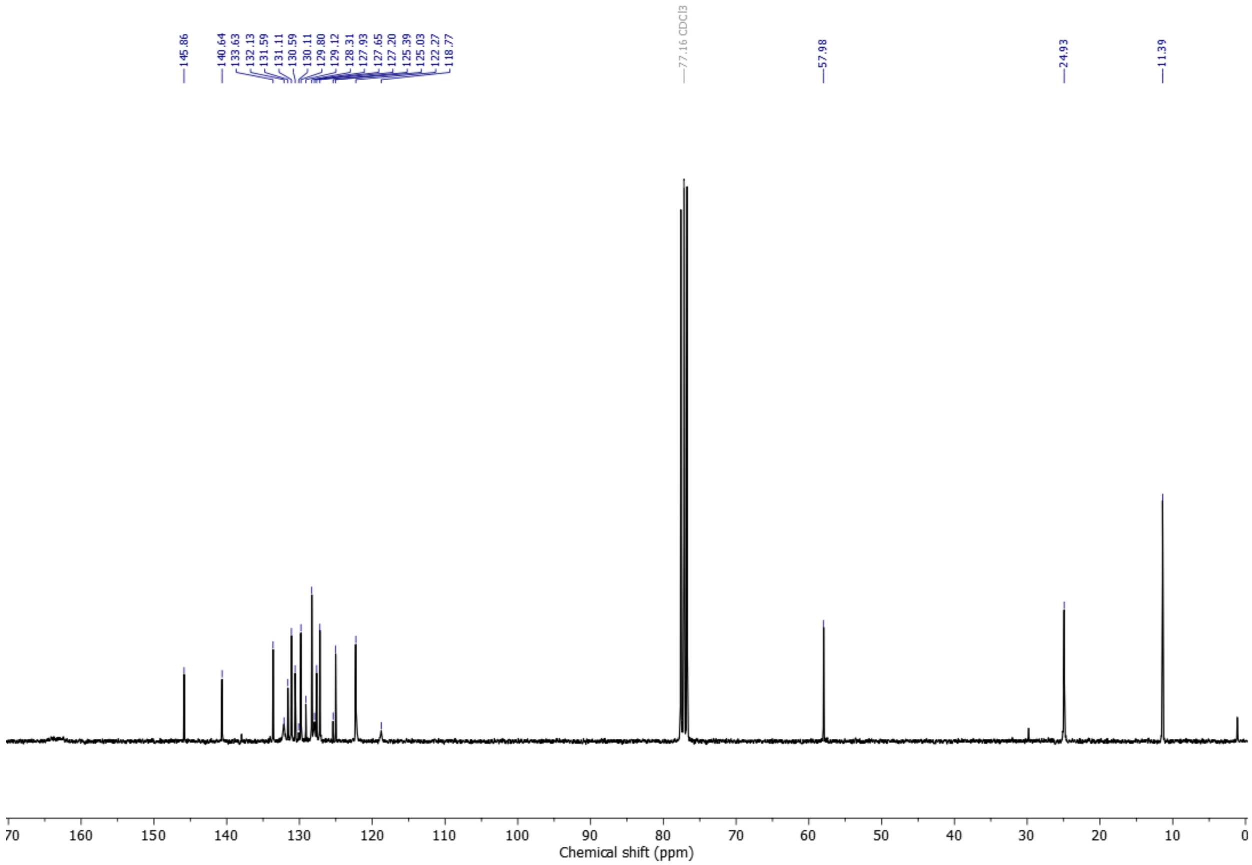

Figure S2. ^{13}C NMR ($CDCl_3$) of **NO_2-BTI**

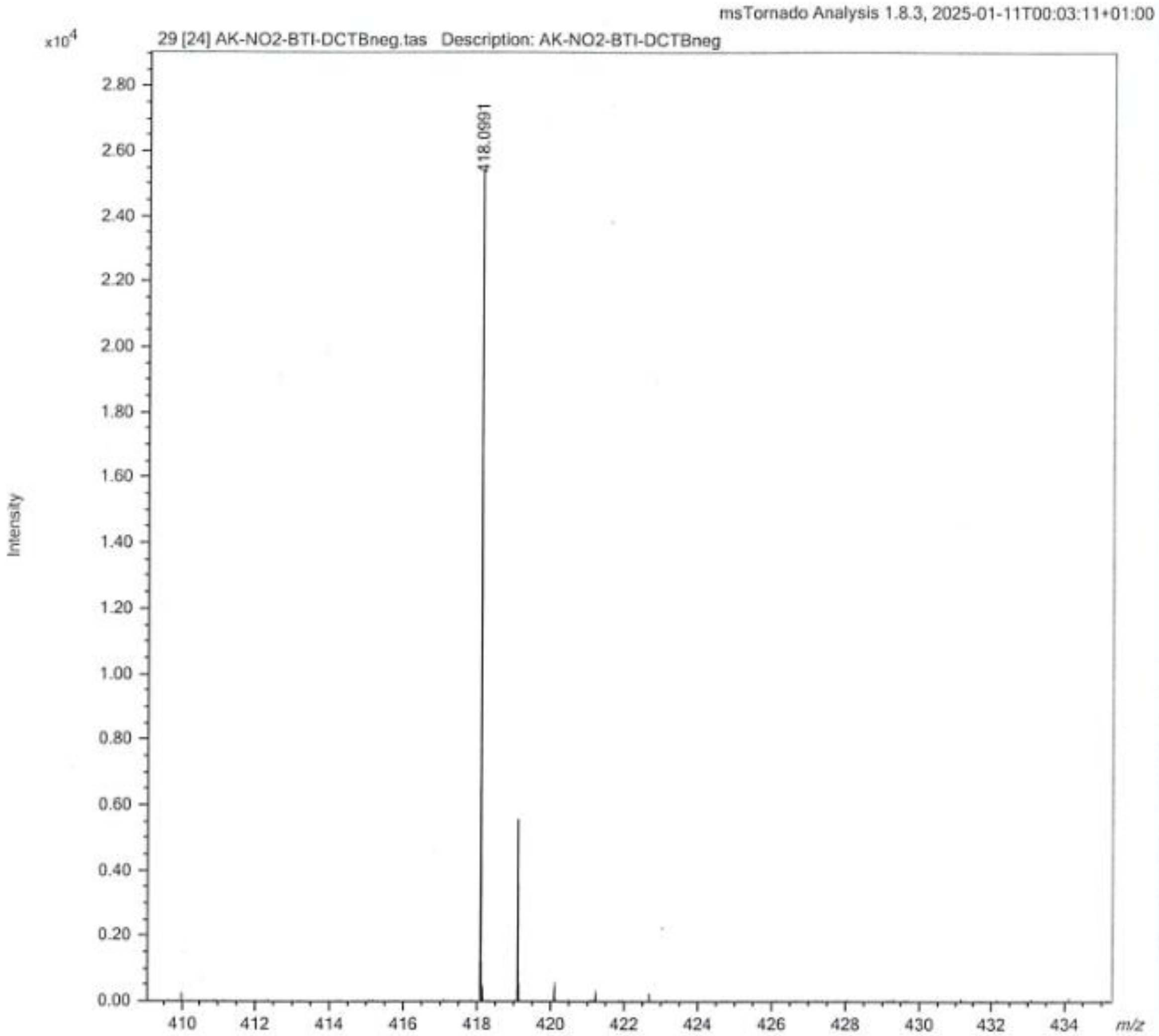

Elemental Composition Estimation

Parameters:

Mass	Tolerance	Electron Mode	Charge	DBE Range	Max Results
418.09914 ± 0.00125	3.0 ppm	Odd	1	-0.5 - 50.0	100

Elements

C 0 - 100 H 0 - 150 N 1 - 5 F 0 - 0 S 1 - 1 O 1 - 10

Results:

#	Formula	Mass	DBE	Abs. Error (u)	Error (u)	Error (ppm)
1	C23 H18 N2 O4 S	418.09928	16.0	0.00013	-0.00013	-0.32

Figure S3. HRMS of **NO_2-BTI**

Synthesis of **TCI-NH**: NO_2-BTI (230 mg, 1 equiv.) and triphenylphosphine (461 mg, 3.2 equiv.) were charged into a flame-dried Schlenk tube equipped with a stir bar. The tube was placed under an argon atmosphere and dry DMF (5 mL) was added and the mixture was bubbled with argon for 10 minutes. The reaction mixture was then stirred at 150 °C for 24 h at which point TLC monitoring showed complete disappearance of the starting NO_2-BTI. The solvent was removed via rotary

evaporation. The crude was then slurred with 5 mL of cold EtOAc. The solids were filtered off and washed with cold EtOAc and a small portion of cold MeOH. After drying under vacuum, **TCI-NH** was obtained as an orange powder (158 mg, 74 % yield).

^{1}H NMR (300 MHz, $CDCl_3$) δ 8.65 (s, 1H), 8.47 (s, 1H), 8.28 (d, J = 8.0 Hz, 1H), 7.51 (d, J = 8.0 Hz, 1H), 7.42 (t, J = 7.9 Hz, 1H), 7.23 (d, J = 8.2 Hz, 1H), 7.06 (d, J = 7.6 Hz, 1H), 5.17 – 5.08 (m, 1H), 2.39 – 2.19 (m, 2H), 1.94 (m, 2H), 0.91 (t, J = 7.5 Hz, 6H).

^{13}C NMR (75 MHz, $CDCl_3$) δ 140.1, 132.7, 130.0, 124.8, 124.0, 124.0, 121.7, 120.7, 119.8, 114.5, 108.8, 31.1, 29.9, 25.2, 11.5, 1.2.

HRMS (MALDI-TOF) calculated for $C_{23}H_{18}N_2O_2S$ 386.10835, found 386.10813 (mass accuracy = -0.57 ppm).

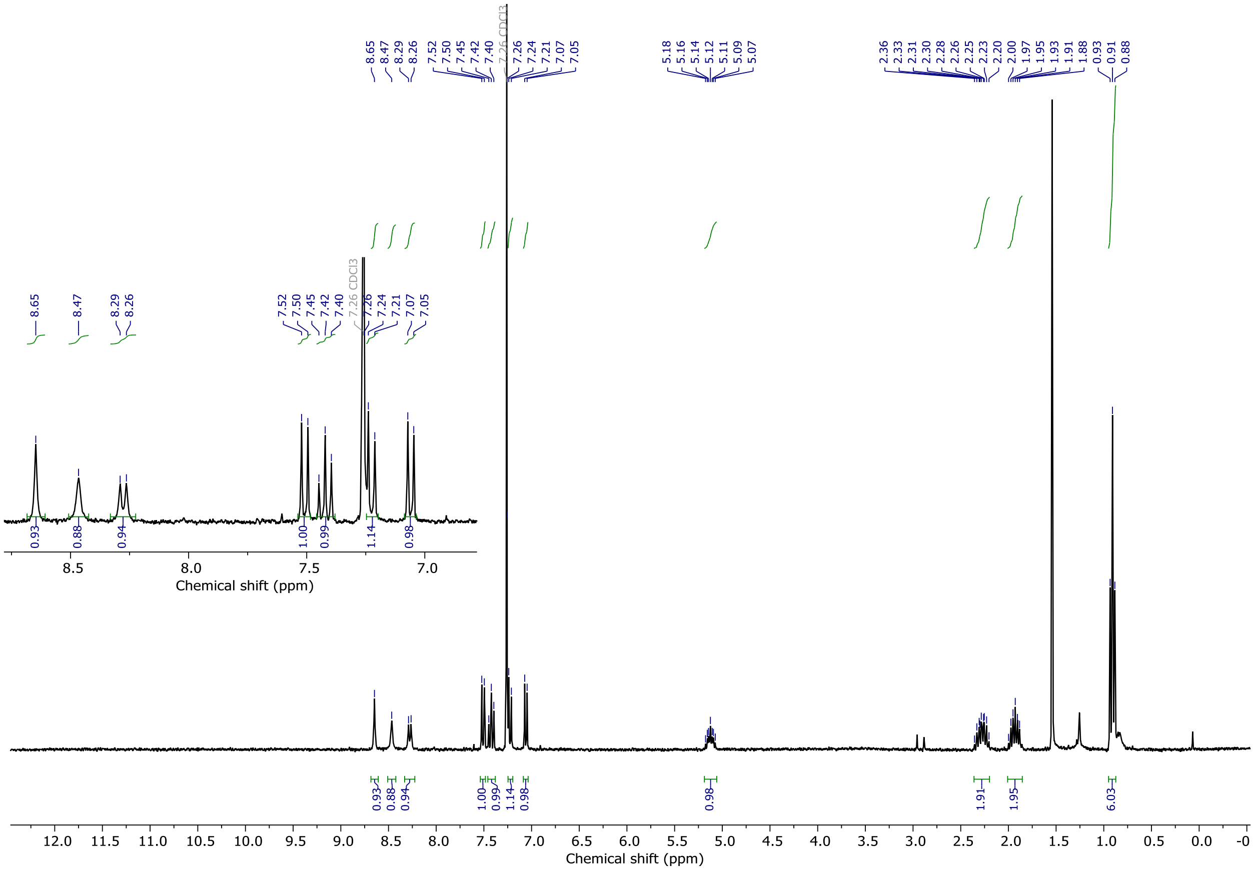

Figure S4. ^{1}H NMR ($CDCl_3$) of **TCI-NH**

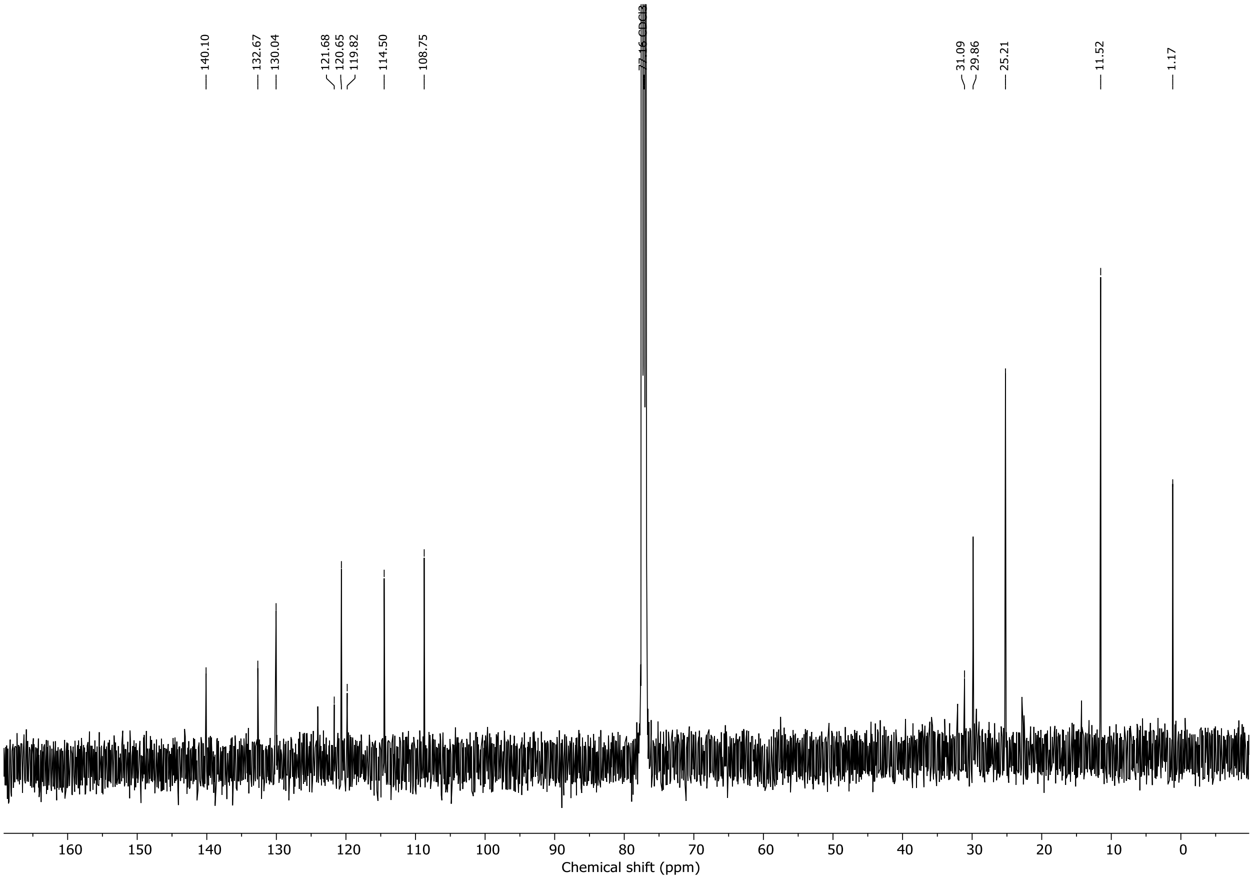

Figure S5. ^{13}C NMR ($CDCl_3$) of **TCI-NH**

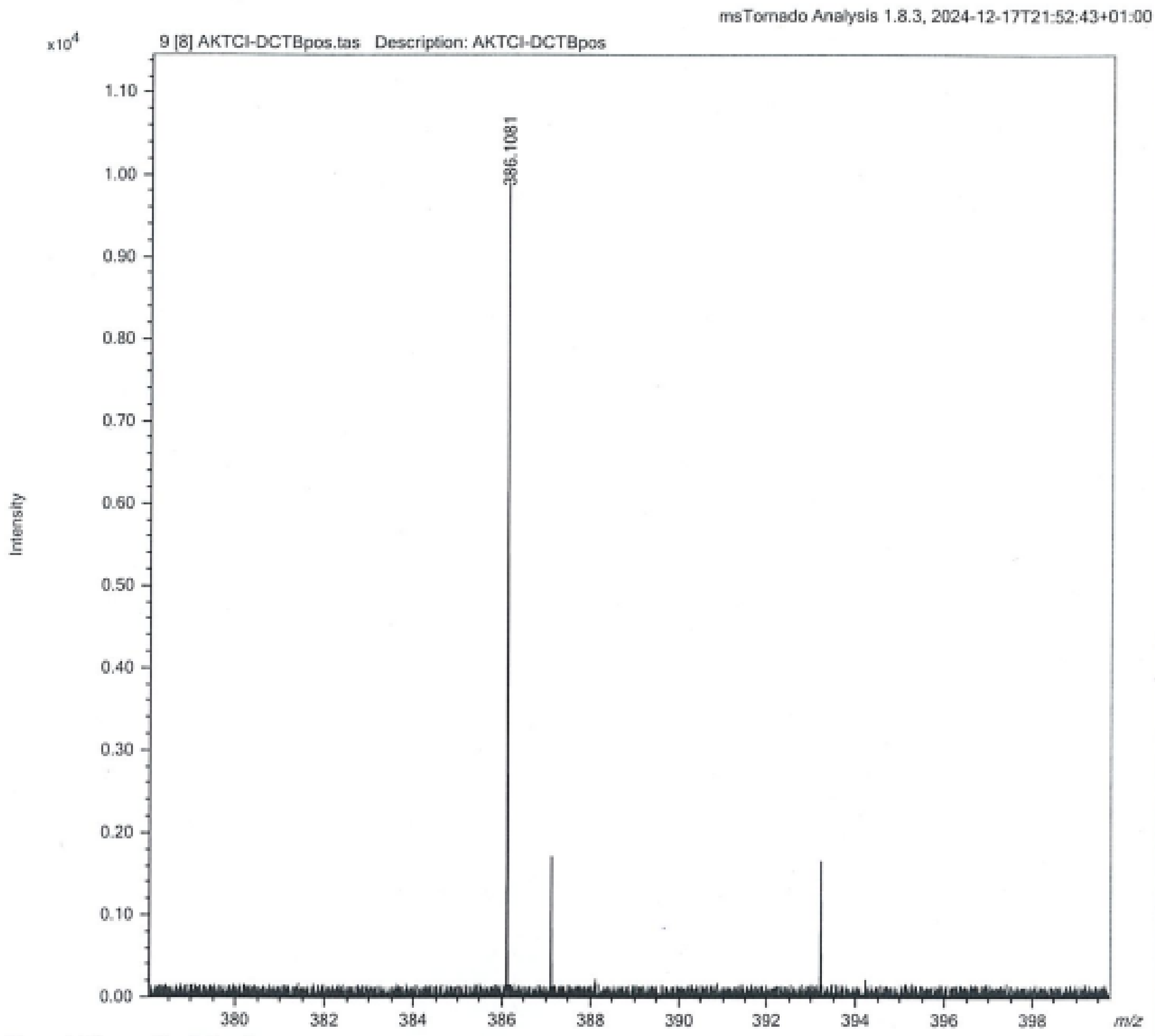

Elemental Composition Estimation

Parameters:

Mass	Tolerance	Electron Mode	Charge	DBE Range	Max Results
386.10813 ± 0.00039	1.0 ppm	Odd	+1	-0.5 - 30.0	100

Elements

C 0 - 70 H 0 - 100 N 1 - 5 O 1 - 5 S 1 - 2

Results:

#	Formula	Mass	DBE	Abs. Error (u)	Error (u)	Error (ppm)
1	C23 H18 N2 O2 S	386.10835	16.0	0.00022	-0.00022	-0.57

Figure S6. HRMS of **TCI-NH**

Crystallographic data

Crystal data were collected on a Rigaku Oxford Diffraction SuperNova diffractometer equipped with an Atlas CCD detector and micro-focus Cu-K_{α} radiation (λ = 1.54184 Å). The structures were solved by dual-space algorithm and refined on F^2 by full matrix least-squares techniques using SHELX package (G.M. Sheldrick, ShelXT2018/2, ShelXL2018/3). All non-hydrogen atoms were refined anisotropically and the H atoms were included in the calculation without refinement. Multiscan empirical absorption was corrected by using CrysAlisPro program (CrysAlisPro, Rigaku Oxford Diffraction, V1.171.41.118a, 2021). Deposition Number(s) 2416213 (for **NO_2-BTI**) and 2416214 (for **TCI-NH**) contain(s) the supplementary crystallographic data for this paper. These data are provided free of charge by the joint Cambridge Crystallographic Data Centre and Fachinformationszentrum Karlsruhe Access Structures service.

Supplementary Table 1. Crystal Data Collection and Refinement Parameters of **TCI-NH**.

Molecule	**TCI-NH**
Empirical formula	$C_{23}H_{18}N_2O_2S$
Formula weight	386.45
Temperature (K)	200.0 (1)
Wavelength(Å)	1.54184
Crystal system, space group	Monoclinic, I 2/a
a(Å)	16.6381(10)
b(Å)	7.2473(5)
c(Å)	30.117(2)
α(deg)	90
β(deg)	104.503(6)
γ(deg)	90
Z	8
Volume (Å^3)	3515.8(4)
Calculated density (Mg/m^3)	1.460
Absorption coefficient (mm^{-1})	1.821
θ range (deg)	3.031 to 73.934
Completeness to θ = 70.000	98.3%
GOF	1.008
Largest diff. peak & hole (e/Å^3)	0.231 and -0.479

Spectroscopy

All spectroscopic measurements were performed on dilute solutions (*ca* 10^{-5} M) in 10 mm quartz cuvettes using spectroscopic grade solvents. Absorption spectra were recorded on a double beam JASCO V-650 spectrometer. Emission spectra were recorded on a Horiba-Jobin-Yvon Fluorolog-3 spectrofluorimeter equipped with a three-slit double grating excitation and emission monochromator with a dispersion of 2.1 nm/mm (1200 groove/mm) in excitation-detection geometry. A R928 photomultiplier was used as a detector. Corrections were applied for both the excitation light intensity variation and the detector spectral response. All fluorescence spectra were corrected for dark counts from the detector. Φ_F were measured in spectroscopic grade chloroform using the relative method. Briefly, emission spectra were acquired for the compound of interest and the reference using the same excitation and detection parameters with an absorbance ranging from 0.1 to 0.01 at the excitation wavelength. The area of each emission curve for the compound x and the reference ref, respectively I_f^x and I_f^{ref}, was plotted as a function of their absorbance (A_x and A_{ref}) to assess the quality of the linear correlation. The Φ_F were calculated according the following equation:

$$\phi_x = \frac{A_{ref} \times I_f^x}{A_x \times I_f^{ref}} \left(\frac{n_x}{n_{ref}}\right)^2 \phi_{ref}$$

here n_x and n_{ref} are the refractive index of the solvent of the compound and reference solutions respectively, Φ_{ref} is the fluorescence quantum yield of the reference, coumarin 153 in Methanol ($\Phi_{ref} = 0.45$). In order to enable use of a similar reference for all compounds discussed in this study (including those reported in prior articles), excitation wavelength was taken at the maximal visible wavelength for each chromophore, and intensity was corrected for wavelength dependent variations of the light source intensity and detector wavelength dependent sensitivity ($S1_c$/ $R1_c$), following the general methodology proposed by Resch-Genger.[2]

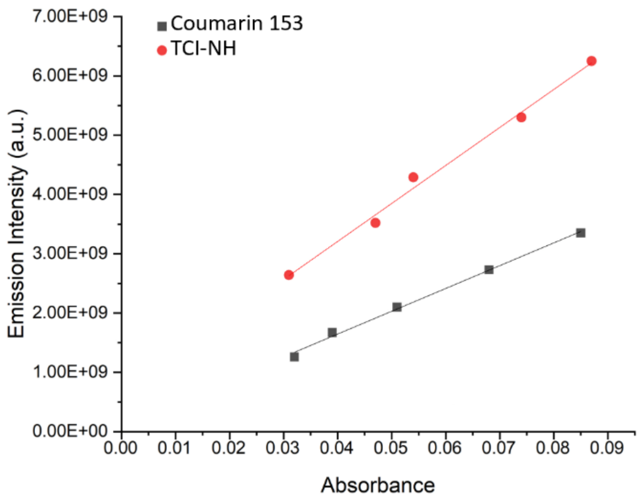

Figure S7. Evolution of the emission intensity (integrated area) *vs* solution absorbance at the excitation wavelength for tested compound **TCI-NH** in chloroform (red dots) and reference compound Coumarin 153 in methanol (black square). Linearization appear as full red and black lines, respectively.

Fluorescence lifetime measurements were performed in chloroform solutions at room temperature on a Horiba-Jobin-Yvon Fluorolog-3 spectrofluorimeter, equipped with a NanoLED 440 operating at 440 nm with 250 ps pulses, an iHR320 emission monochromator with 1200 groves mm-1 gratings and a R928 photomultiplier. To obtain the Instrument Response Function, a highly diffusing colloidal silica solution (Ludox®) from Sigma-Aldrich diluted in water was used. Fluorescence lifetimes were determined by deconvolution of the acquired signal using the decay analysis software (DAS).

Φ_{Δ} were measured by the relative methodology, through evaluation of the singlet oxygen phosphorescence intensity of diluted chloroform solutions of the PS. Singlet-oxygen phosphorescence was recorded on a Horiba-Jobin-Yvon Fluorolog-3 spectrofluorimeter in excitation-detection geometry using a liquid nitrogen cooled, solid indium/gallium/arsenide (InGaAs) detector, operating in the range 850–1600 nm. The singlet oxygen generation quantum yield $\Phi_{\Delta x}$ was measured in the range 1220-1320 nm in spectroscopic grade chloroform solutions with an optical density lower than 0.1 under stirring. Reference was Phenalenone in spectroscopic grade chloroform ($\Phi_{\Delta ref}$ = 0.95). Signal was acquired upon excitation of the chromophore at its absorption maximum and signal intensity was corrected by dividing the signal intensity by the lamp power, to account for wavelength dependent intensity variations (T_{1c}/R_{1C}) $\Phi_{\Delta x}$ was calculated according to the following equation:

$$\phi_{\Delta x} = \frac{A_{ref} \times \ I_{\Delta}^{x}}{A_x \times \ I_{\Delta}^{ref}} \phi_{\Delta ref}$$

Where I_{Δ}^{x} and I_{Δ}^{ref} stand for the lamp, detector and dark offset corrected intensity of the singlet oxygen phosphorescence of the sample and reference, respectively, A_x and A_{ref} for the absorbance of the sample and reference at their maximal absorption wavelength[2] used for the excitation, respectively, $\Phi_{\Delta ref}$ is the singlet oxygen quantum yield of the reference, phenalenone in chloroform ($\Phi_{\Delta ref}$ = 0.95).

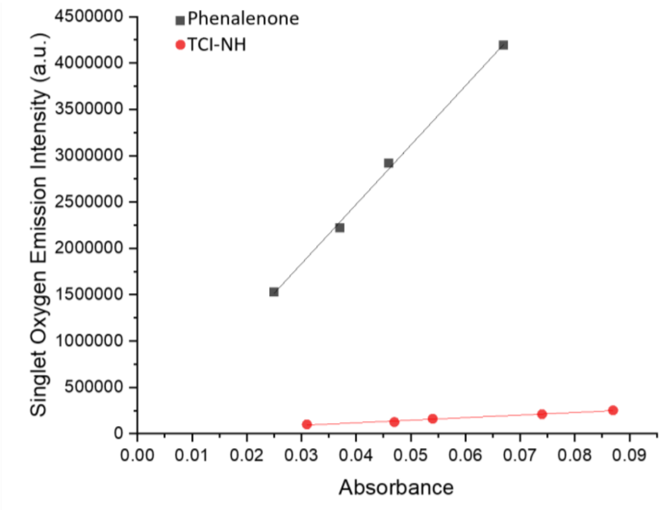

Figure S8. Evolution of the singlet oxygen emission intensity @1270 nm (integrated area) *vs* solution absorbance at the excitation wavelength for tested compound **TCI-NH** in chloroform (red dots) and reference compound phenalenone in chloroform (black square). Linearization appear as full red and black lines, respectively.

TCI-NH, DNA and Protein Preparation

A 5 mM stock solution of **TCI-NH** was prepared by dissolving the compound in DMSO. This solution was used for photosensitization studies in aqueous systems—including experiments both with and without biological matrices—as well as *in cellulo* assays. DMSO was selected not only to ensure high solubility but also to minimize aggregation. In subsequent biological assays, the DMSO concentration was carefully limited to 0.5% (v/v), a threshold that our cell culture studies have consistently shown to be non-cytotoxic.[3-5] Bovine serum albumin (BSA, Sigma-Aldrich) and DNA (Sigma-Aldrich) stock solutions were prepared in a 50 mM Tris-phosphate buffer (pH 6.8) supplemented with 100 mM KCl. DNA samples were heated at 95 °C for 5 minutes and then allowed to equilibrate at room temperature overnight to promote G4 folding. Oligonucleotide concentrations were determined using a JASCO V-730 spectrometer, applying molar extinction coefficients at 260 nm calculated with the OligoAnalyzer web tool (Integrated DNA Technologies). The following extinction coefficients were used: *c-MYC* G4 DNA (5′-TGAGGGTGGGTAGGGTGGGTAA-3′), $\varepsilon_{260\ nm}$ = 228,700 M^{-1} cm^{-1}; ssDNA (5′-CGCGCTCCCGCCCCCTCTCCCCTCCCCGCGC-3′), $\varepsilon_{260\ nm}$ = 238,500 M^{-1} cm^{-1}; dsDNA (5′-CAATCGGATCGAATTCGATCCGATTG-3′), $\varepsilon_{260\ nm}$ = 253,200 M^{-1} cm^{-1}. All optical measurements were performed in quartz cuvettes with a 10 mm path length.

Fluorescence Titration Binding Studies

Emission titrations were performed by maintaining a fixed concentration of **TCI-NH** (2.5 μM) in a 50 mM Tris-phosphate buffer (pH 6.8) supplemented with 100 mM KCl at 25 °C. BSA or DNA structures were then added in concentrations ranging from 0 to 15 μM. After each addition, the mixture was allowed to equilibrate for 5 minutes before measurement. Fluorescence spectra were recorded using a JASCO FP-8550 fluorimeter equipped with a Julabo CD-B5 cooling system. The samples were excited at 488 nm (corresponding to the absorption maximum in the buffered aqueous solution of **TCI-NH**), and the emission was collected from 495 to 750 nm. Emission spectra are presented as the fold-change in fluorescence intensity, determined by dividing the emission maximum of the free **TCI-NH** by the emission maxima observed upon incremental addition of the biological templates. This approach isolates the contribution of template binding to the fluorescence enhancement. Quantitative analysis to derive association constants (K_a) was performed using nonlinear global fitting with the Bindfit web-based tool.

ROS Generation Assays

Dihydrorhodamine-123 (DHR-123, MedChemExpress) (5 μM) served as the $O_2^{\bullet-}$ sensor. Samples containing **TCI-NH** (5 μM), either alone or complexed with *c-MYC* G4 DNA or BSA (10 μM), were prepared in a 50 mM Tris-phosphate buffer (pH 6.8) supplemented with 100 mM KCl. Each solution was irradiated using a Hamamatsu Photonics L9588-04 light source equipped with a 485 ± 20 nm bandpass filter. Irradiation intervals ranged from 0 to 300 seconds. After each interval, the emission of DHR-123 (excitation at 488 nm) was recorded using a JASCO FP-8550 fluorimeter. Baseline contributions from all components were subtracted to isolate the fluorescence attributable solely to $O_2^{\bullet-}$ generation. Under these optimized conditions, self-activation of DHR-123 by light is negligible.

9,10-anthracenediyl-bis(methylene)dimalonic acid (ABDA, MedChemExpress) (30 μM) was employed as the $^1O_2$ sensor. Samples containing **TCI-NH** (5 μM), either alone or complexed with *c-MYC* G4 DNA or BSA (10 μM), were prepared under the same buffer conditions. Irradiation was performed using the same light source and filter, with time points ranging from 0 to 600 seconds. After each interval, the absorption spectrum of ABDA was recorded using a JASCO V-730 spectrometer. Contributions from all additives were accounted for in the reference cuvette, ensuring that any observed ABDA bleaching directly reflected $^1O_2$ production. Under these conditions, ABDA self-bleaching is negligible.

Φ_Δ for **TCI-NH**, **TCI-NH**–G4 DNA, and **TCI-NH**–BSA were determined using methylene blue (MB) as a reference (Φ_Δ(MB) = 0.52 in water). The kinetics of ABDA bleaching followed a first-order decay, allowing for linear regression analysis. The Φ_Δ were calculated by integrating the absorption band within the 465–505 nm range and comparing the bleaching rates and integrated absorbances between the sample (*i.e.* **TCI-NH**, **TCI-NH**-G4 DNA or **TCI-NH**-BSA) and MB:

$$\Phi_{\Delta(sample)} = \Phi_{\Delta(MB)} \times \frac{k_{sample}}{k_{MB}} \times \frac{\int_{465}^{505} A_{MB}(\lambda)d\lambda}{\int_{465}^{505} A_{sample}(\lambda)d\lambda}$$

where k_{sample} and k_{MB} are the rate constants for ABDA bleaching in the presence of the sample and MB, respectively, and the integrals represent the integrated absorbance of the MB and sample over the specified wavelength range.

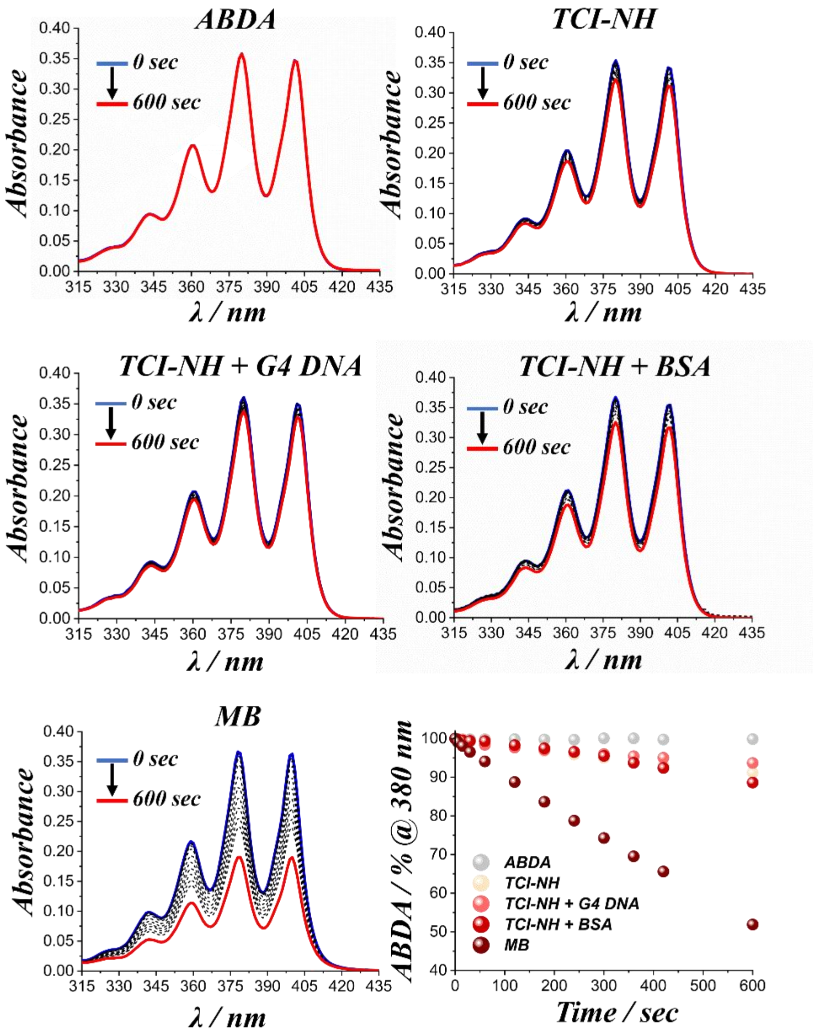

Figure S9. Detection of $^1O_2$ using ABDA as a sensor. Bleaching of ABDA (30 µM) was monitored as an indicator of $^1O_2$ generation by **TCI-NH** (5 µM) or MB (5 µM) upon irradiation. Samples were irradiated at various time intervals (0–600 seconds) using excitation at 485 ± 20 nm. **TCI-NH** experiments were conducted in the presence of BSA (10 µM) or G4 DNA (10 µM) in Tris-HCl buffer (50 mM, pH 6.8) with 100 mM KCl. MB photoactivation was performed in distilled water.

Intracellular ROS Generation

10×10^4 HeLa cells were seeded one day prior to treatment onto glass-bottom microwell dishes (MatTek Corp.). The following day, the cells were treated with **TCI-NH** at a concentration of 0.5 μM or with an equivalent volume of DMSO (0.04% v/v) as a control and then incubated at 37 °C in a 5% CO_2 atmosphere for 24 hours. When required, the cells were photo-irradiated using an EVOS® FL cell imaging system equipped with an adjustable-intensity LED cube (excitation wavelength: 470 ± 22 nm) operating at an intensity of 27 mW/cm^2 for 6 minutes. After irradiation, CellROX Green Reagent (Invitrogen) was added to the cells at a final concentration of 5 μM and incubated for 30 minutes at 37 °C in 5% CO_2 to detect ROS. Following incubation, the cells were washed twice with 1×PBS to remove excess reagent before being fixed with 4% paraformaldehyde (PFA). Prior to imaging, the fixed cells were incubated with Hoechst 33258 at a concentration of 500 nM and subsequently washed three times with 1×PBS to remove unbound dye. Fluorescent images were acquired using a Leica SP8 FALCON confocal microscope, and maximum intensity projections of Z-stack images were utilized for data presentation. All imaging data were processed and analyzed using ImageJ software.

(Photo)Toxicity Investigations

HeLa cells were seeded at a density of 5×10^3 cells per well in 96-well plates one day prior to treatment with **TCI-NH**. **TCI-NH** was dissolved in complete medium at varying concentrations, ensuring that the final concentration of DMSO did not exceed 0.5% (v/v), and subsequently added to the cells. The cells were then incubated at 37 °C in a 5% CO_2 atmosphere for 24 hours. When indicated, the treated cells underwent photo-irradiation using an EVOS® FL Cell Imaging System equipped with a LED cube set to an excitation wavelength of 470 ± 22 nm and operating at an intensity of 27 mW/cm^2 for 6 minutes. Following photo-irradiation, the cells were further incubated for an additional 24 hours under the same conditions (37 °C, 5% CO_2). At 48 hours post-initiation of **TCI-NH** treatment, PrestoBlue™ reagent (Invitrogen) was added to each well, and the cells were incubated for an additional three hours at 37 °C in a 5% CO_2 environment. Cell viability was assessed by measuring the fluorescence signal emitted by PrestoBlue™ (excitation/emission wavelengths: 560/590 nm) using a Synergy H4 microplate reader (BioTek).

Live Cell Imaging via Confocal Microscopy

One day prior to treatment, a total of 1×10^4 HeLa cells were plated on glass-bottom microwell dishes (MatTek Corp.) in DMEM medium supplemented with 1% penicillin–streptomycin and 10% fetal bovine serum. The cells were maintained at 37 °C in a 5% CO_2 atmosphere. HeLa cells were washed twice with DMEM medium and then treated with **TCI-NH** (500 nM) for 24 hours. After treatment, the cells were washed twice with 1×PBS before being incubated with organelle-selective stains. Nuclear staining was performed using Hoechst 33342 (500 nM; Sigma-Aldrich), while ER-Tracker Red (500 nM; Thermo Fisher Scientific), Lyso-Tracker Red (100 nM; Thermo Fisher Scientific), or Mito Tracker Red (100 nM; Thermo Fisher Scientific) were utilized for organelle-specific staining. These dyes were dissolved in a live cell imaging solution (Molecular

Probes) and incubated with the cells for 30 minutes at 37 °C in a 5% CO_2 environment. Prior to imaging, the cells were washed twice with the live cell imaging solution to remove any unbound dyes. Imaging was conducted using a Leica SP8 FALCON confocal microscope equipped with an incubation chamber set to maintain 37 °C and 5% CO_2. Maximum intensity projections of Z-stack images were generated for data presentation. All imaging data were subsequently processed using ImageJ software.

Dynamic Imaging of Morphological Changes

One day prior to treatment, 5×10^3 HeLa cells were seeded per well in 96-well plates containing complete medium. **TCI-NH** at a concentration of 1 μM, or an equivalent volume of DMSO (0.08% v/v) as a control, were dissolved in complete medium and added to the cells. The cells were then incubated for an additional 24 hours at 37 °C in a 5% CO_2 atmosphere. Subsequently, the treated cells were subjected to photo-irradiation using an EVOS® FL Cell Imaging System equipped with adjustable-intensity LED cubes. The irradiation was performed at an excitation wavelength of 470 ± 22 nm and an intensity of 30 mW/cm^2.

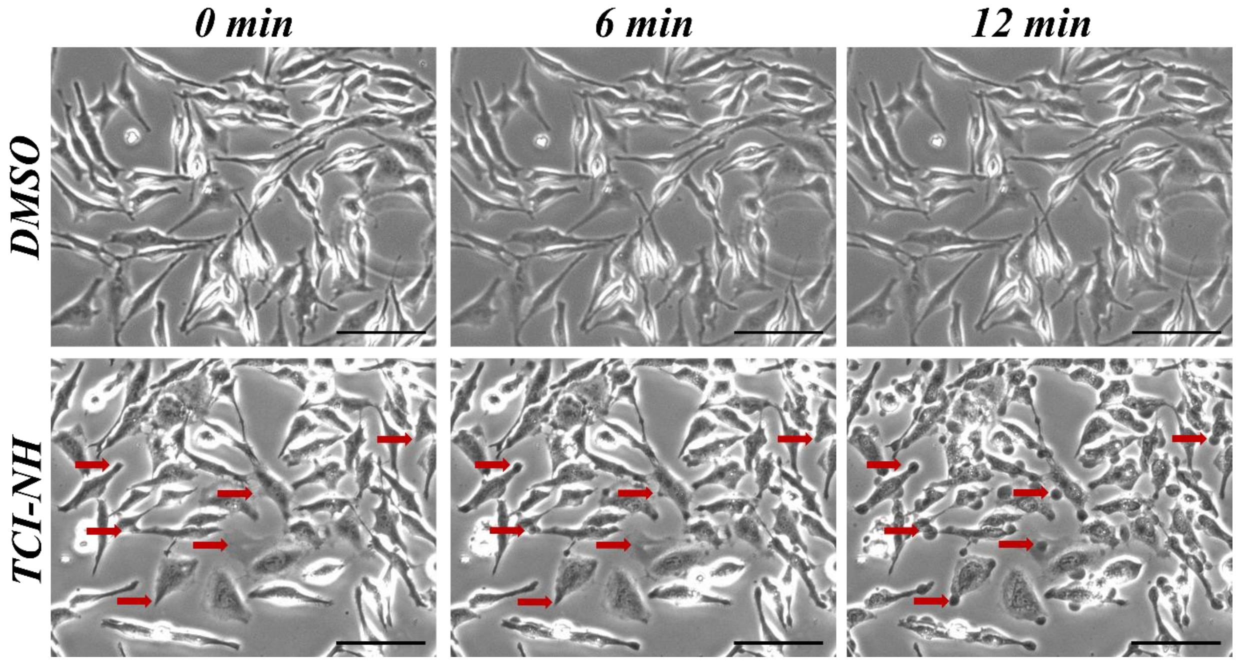

Figure S10. Time-lapse analysis of light-induced morphological changes in HeLa cells. Time-lapse experiments were performed to observe morphological alterations in HeLa cells treated with **TCI-NH** (1 μM) under blue light irradiation. Images were captured at 0, 6, and 12 minutes post-irradiation. Control experiments were conducted using HeLa cells treated with DMSO under the same conditions. Red arrows indicate the formation of apoptotic bodies during the light exposure. Scale bar: 100 μm.

EPR analyses

TEMP (Sigma-Aldrich, 97%) and DMPO (TCI chemicals, 97%) samples were prepared in air atmosphere, as 5 • 10^{-3} M solutions in chloroform and DMSO, respectively. 2 • 10^{-4} M DMSO and chloroform solutions of **TCI-NH**, DBI and BTI were also prepared. 100 µL of each PS and scavenger solutions (3 samples in dichloromethane and three in DMSO) were mixed immediately before EPR experiments, and introduced into capillary tubes, which were protected from light by an aluminum foil up to the beginning of the irradiation. The irradiation (0.7-17.5 min) was performed using a ThorlabLEDs (405-455 nm) directly into the EPR cavity while the spectrum is recording. EPR assays were all carried out at room temperature using a Bruker E500 spectrometer operating at X-band (9.44GHz), with 100 KHz modulation frequency. The instrument settings were as follows: microwave power; 2-69 mW; modulation amplitude; 1 G; Hyperfine coupling constants a and g values were obtained with simulation of experimental spectra using easyspin (Matlab toolbox).

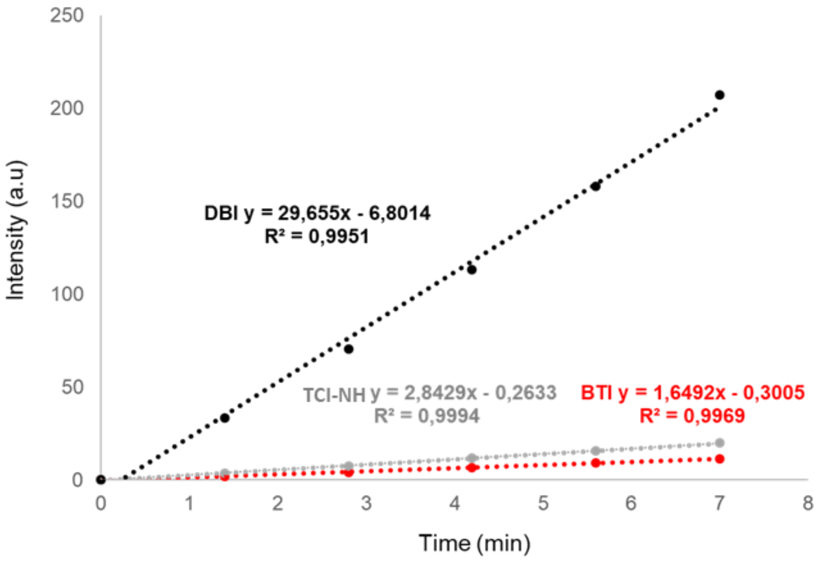

Figure S11. Temporal evolution of TEMPO signal intensity upon irradiation of a solution of TEMP and DBI (black dots), BTI (red dots) and **TCI-NH** (grey dots), and associated linearization to pseudo zero-order kinetics (black, red, and grey dotted lines, respectively).

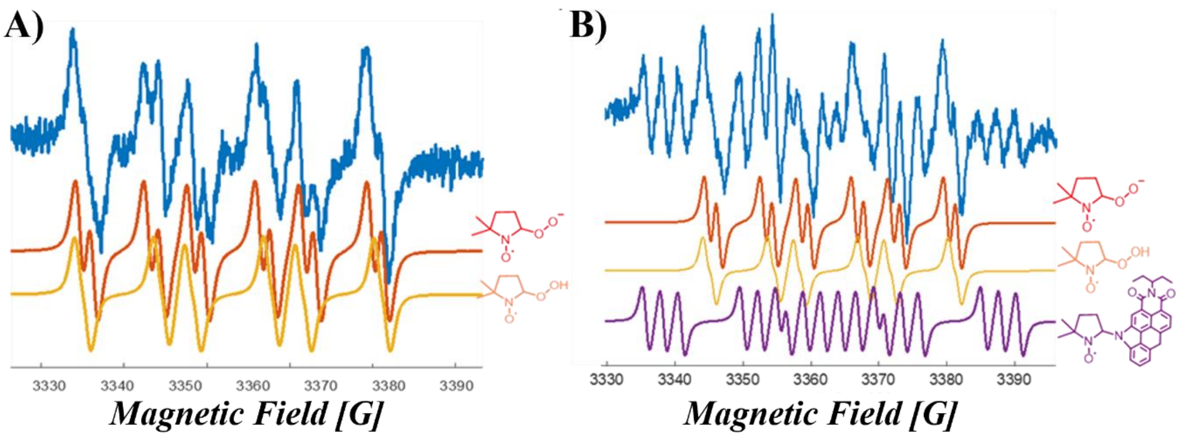

Figure S12. Experimental spectra (blue) of irradiated DMSO solutions of **A)** BTI and **B) TCI-NH** using DMPO as a radical scavenger, and signatures of the different DMPO adducts with $O_2^{\bullet-}$ (red), HOO$^{\bullet}$ (orange), and TCI-N$^{\bullet}$ (purple) identified by deconvolution of the experimental signal.

References:

(1) Josse, P.; Morice, K.; Puchán Sánchez, D.; Ghanem, T.; Boixel, J.; Blanchard, P.; Cabanetos, C. Revisiting the synthesis of the benzothioxanthene imide five decades later. *New Journal of Chemistry* **2022**, *46* (18), 8393-8397, 10.1039/D2NJ00955B.

(2) Würth, C.; Grabolle, M.; Pauli, J.; Spieles, M.; Resch-Genger, U. Comparison of Methods and Achievable Uncertainties for the Relative and Absolute Measurement of Photoluminescence Quantum Yields. *Analytical Chemistry* **2011**, *83* (9), 3431-3439.

(3) Deiana, M.; Josse, P.; Dalinot, C.; Osmolovskyi, A.; Marqués, P. S.; Castán, J. M. A.; Abad Galán, L.; Allain, M.; Khrouz, L.; Maury, O.; et al. Site-selected thionated benzothioxanthene chromophores as heavy-atom-free small-molecule photosensitizers for photodynamic therapy. *Communications Chemistry* **2022**, *5* (1), 142.

(4) Deiana, M.; Andrés Castán, José M.; Josse, P.; Kahsay, A.; Sánchez, Darío P.; Morice, K.; Gillet, N.; Ravindranath, R.; Patel, Ankit K.; Sengupta, P.; et al. A new G-quadruplex-specific photosensitizer inducing genome instability in cancer cells by triggering oxidative DNA damage and impeding replication fork progression. *Nucleic Acids Research* **2023**, *51* (12), 6264-6285.

(5) Sánchez, D. P.; Morice, K.; Mutovska, M. G.; Khrouz, L.; Josse, P.; Allain, M.; Gohier, F.; Blanchard, P.; Monnereau, C.; Le Bahers, T.; et al. Heavy-atom-free π-twisted photosensitizers for fluorescence bioimaging and photodynamic therapy. *Journal of Materials Chemistry B* **2024**, *12* (33), 8107-8121.
