# Supplementary material for: Organelle-Specific Thiochromenocarbazole Imide Derivative as a Heavy-Atom-Free Type I Photosensitizer for Biomolecule-Triggered Image-Guided Photodynamic Therapy
Source: J Phys Chem Lett. 2025 Feb 24;16(9):2273–82. doi: 10.1021/acs.jpclett.5c00136 (PMC11891978; doi:10.1021/acs.jpclett.5c00136)
Supplement: Supplementary file 2 — jz5c00136_si_002.pdf [file jz5c00136_si_002.pdf]

jz-2025-001362.R1

Name: Peer Review Information for "Organelle-Specific Thiochromenocarbazole Imide Derivative as a Heavy-Atom-Free Type I Photosensitizer for Biomolecule-Triggered Image-Guided Photodynamic Therapy"

First Round of Reviewer Comments

Reviewer: 1

Comments to the Author

The Manuscript “Organelle-Specific Thiochromenocarbazole Imide Derivative

as a Heavy-Atom-Free Type I Photosensitizer for Biomolecule-Triggered Image-Guided Photodynamic Therapy”, ID jz-2025-001362 submitted to The Journal of Physical Chemistry Letters by Pierre Josse, Clément Cabanetos and Marco Deiana as corresponding authors, introduces a new TCI-NH compound, a thiochromenocarbazole imide derivative, as a next-generation, heavy-atom-free photosensitizer (PS) for type I photodynamic therapy (PDT).

The study employs a robust combination of spectroscopic, photophysical, and biological assays to demonstrate the efficacy of TCI-NH. This includes fluorescence titration, ROS detection, cellular imaging, and phototoxicity studies.

Unlike traditional type II PDT relying on singlet oxygen (1O2), TCI-NH primarily generates superoxide radicals (O2•–) and PS-centered radicals. TCI-NH exhibits high luminescence efficiency and selectively targets organelles such as the endoplasmic reticulum (ER) and mitochondria, key sites for apoptotic signalling. Its fluorescence increases significantly in the presence of biomolecules like bovine serum albumin (BSA) or G-quadruplex DNA (G4 DNA), enhancing ROS generation up to 5-fold.

TCI-NH compound demonstrates excellent fluorescence with a quantum yield of ~0.88 and a long excited-state lifetime of 7.3 ns. Moreover, Confocal imaging shows selective accumulation in ER and mitochondria, with minimal nuclear or lysosomal localization.

Under light activation, TCI-NH exhibits high photocytotoxicity (IC50 ~120 nM) with minimal dark toxicity and a phototherapeutic index exceeding 200. It operates via a type I PDT mechanism, inducing apoptosis through mitochondrial and ER pathways.

This research highlights a promising paradigm shift in PDT by leveraging type I mechanisms over type II, overcoming limitations like oxygen dependency and off-target effects. Using a heavy-atom-free platform aligns with contemporary efforts to enhance biocompatibility and reduce dark toxicity. Notably, the study demonstrates an impressive phototherapeutic index and rapid induction of apoptotic cell death, positioning TCI-NH as a highly effective and safer alternative to traditional photosensitizers. I am thrilled to accept this manuscript after minor changes.

Minor suggestions:

1.- The study primarily focuses on in vitro experiments, necessitating further validation in vivo. Are the authors planning to proceed with these studies? A sentence is necessary for the conclusions related to this topic.

2.- Direct comparisons with other state-of-the-art photosensitizers (e.g., in terms of efficiency, cost of synthesis, or stability under physiological conditions) are underexplored and must be reported in the introduction.

3.- As the interactions of TCI-NH with biomolecules, such as DNA and proteins, were demonstrated, more deep structural insights (e.g., through docking studies or crystallography) are missing. These would better elucidate binding modes and affinities. I suggest the authors include a paragraph related to this topic in the discussion.

4.- Finally, the authors have only tested HeLa cells. Expanding the studies to other cancer cell lines or 3D tumour spheroids in a future paper could better demonstrate the generalizability of findings.

Reviewer: 2

Comments to the Author

This manuscript reports about a very interesting and efficient PDT type I photosensitizer, which was tested on HeLa cells with very strong radical formation and cell death induction at relatively low concentrations and upon irradiation with blue light for relatively short time periods. Control

experiments show that there the dark toxicity is negligible. Various characterization techniques (including extraterrestrial power radiation – EPR) were used to carefully analyze the cellular toxicity results. The PDT effect of the TCI-NH agent is impressive, the study is original and novel, and the results are interesting and impactful for the physical chemistry community. Unfortunately, the manuscript lacks clarity sometimes and many of my comments below may have been caused by misunderstanding of the experimental conditions and/or the results. I suggest that the authors clarify those points, which can probably be accomplished by minor but careful revision.

Comments:

Figure 2: Why were absorption, emission, and quantum yields measured in chloroform? And for cell imaging the TCI-NH was also in chloroform? It is not clear because only the concentrations are provided and a certain amount of DMSO is mentioned. Before the cell experiments, the TCI-NH should be analyzed in cell culture medium or other aqueous media.

In fact, the following paragraphs mention that TCI-NH emits around 580 nm in aqueous. That means that the green signal in the imaging experiments in Figure 3 (495–560 nm) are probably not related to TCI-NH. On the other hand, interaction with proteins shifts the fluorescence back to the blue (527 nm) which means the green channel is maybe related to TCI-NH emission. I think spectroscopic analysis of TCI-NH in cell culture medium or better in a solution containing also cells, would be important.

The experiments concerning the non-production of singlet oxygen could be moved to the SI and the manuscript (if it remains in letter type) could simply mention that singlet oxygen production was negligible.

ROS production experiments were again performed in chloroform?

Figure 5B: How can the authors be sure that the green emission is CellROX because TCI-NH is also green (at least in the initial imaging experiments)? Maybe it is just TCI-NH emission and not ROS production?

Figure 6B: With such a strong phototoxicity, would one not expect that the irradiated cells in Figure 5B would look a little more damaged? They still look pretty healthy in those images. It would be great to specify the conditions in the figure captions, such that the results are comparable. Now one has to go back and forth between the manuscript text, the experimental part, and the figures to

understand the different conditions (such as incubation times, solvents etc.). And, in fact, the cells do deform (Figure S10) but not in Figure 5B.

Figure S10: Why is DMSO (and not chloroform) used as control solution?

Abbreviations EPR, TEMPO, DMPO, and TEMP wni (were not introduced). Why again solvent change (chloroform in the main experiments - I assume? – and DMSO in the controls)?

Author's Response to Peer Review Comments:

Assistant Professor
Marco Deiana
m.deiana@pwr.edu.pl

Dear Editor,

We are grateful for the opportunity to resubmit our revised research article titled *“Organelle-Specific Thiochromenocarbazole Imide Derivative as a Heavy-Atom-Free Type I Photosensitizer for Biomolecule-Triggered Image-Guided Photodynamic Therapy”* (Manuscript ID: jz-2025-001362) for consideration in **The Journal of Physical Chemistry Letters**. We sincerely thank you and the reviewers for the constructive feedback, which has greatly enhanced the quality of our work.

Both reviewers provided positive evaluations of our work, recommending only minor revisions. In this revised version, we have thoroughly addressed all comments and suggestions, clarifying aspects that required further explanation. To maintain the concise focus appropriate for a letter-format article, we have kept the discussion targeted.

We have also addressed the formatting requirements for the Table of Contents graphic, which is now included within the manuscript text.

The revised manuscript has been submitted in two versions: one with tracked changes highlighting all modifications and a clean version without markup. We trust that these revisions address all concerns raised and meet the high standards of **The Journal of Physical Chemistry Letters**. We look forward to your positive feedback and remain available to provide any further information or clarification if needed.

Thank you once again for considering our manuscript for publication.

Sincerely,

Marco Deiana Clément Cabanetos

POINT-BY-POINT RESPONSE TO REVIEWERS' COMMENTS

(responses in blue, changed text in red)

REVIEWER REPORTS

Reviewer: 1

Recommendation: This paper is publishable subject to minor revisions noted. Further review is not needed.

Comments:

The Manuscript “Organelle-Specific Thiochromenocarbazole Imide Derivative as a Heavy-Atom-Free Type I Photosensitizer for Biomolecule-Triggered Image-Guided Photodynamic Therapy”, ID jz-2025-001362 submitted to The Journal of Physical Chemistry Letters by Pierre Josse, Clément Cabanetos and Marco Deiana as corresponding authors, introduces a new TCI-NH compound, a thiochromenocarbazole imide derivative, as a next-generation, heavyatom-free photosensitizer (PS) for type I photodynamic therapy (PDT).

The study employs a robust combination of spectroscopic, photophysical, and biological assays to demonstrate the efficacy of TCI-NH. This includes fluorescence titration, ROS detection, cellular imaging, and phototoxicity studies.

Unlike traditional type II PDT relying on singlet oxygen (1O2), TCI-NH primarily generates superoxide radicals (O2•–) and PS-centered radicals. TCI-NH exhibits high luminescence efficiency and selectively targets organelles such as the endoplasmic reticulum (ER) and mitochondria, key sites for apoptotic signalling. Its fluorescence increases significantly in the presence of biomolecules like bovine serum albumin (BSA) or G-quadruplex DNA (G4 DNA), enhancing ROS generation up to 5-fold.

TCI-NH compound demonstrates excellent fluorescence with a quantum yield of ~0.88 and a long excited-state lifetime of 7.3 ns. Moreover, Confocal imaging shows selective accumulation in ER and mitochondria, with minimal nuclear or lysosomal localization.

Under light activation, TCI-NH exhibits high photocytotoxicity (IC50 ~120 nM) with minimal dark toxicity and a phototherapeutic index exceeding 200. It operates via a type I PDT mechanism, inducing apoptosis through mitochondrial and ER pathways.

This research highlights a promising paradigm shift in PDT by leveraging type I mechanisms over type II, overcoming limitations like oxygen dependency and off-target effects. Using a heavy-atom-free platform aligns with contemporary efforts to enhance biocompatibility and reduce dark toxicity. Notably, the study demonstrates an impressive phototherapeutic index and rapid induction of apoptotic cell death, positioning TCI-NH as a highly effective and safer alternative to traditional photosensitizers. I am thrilled to accept this manuscript after minor changes.

Minor suggestions:

1.- The study primarily focuses on in vitro experiments, necessitating further validation in vivo. Are the authors planning to proceed with these studies? A sentence is necessary for the conclusions related to this topic.

- We thank the Reviewer for this valuable suggestion. In response, we have revised the manuscript to include the following statement in the conclusions:

To further advance the therapeutic outcome and accelerate clinical translation, we have initiated studies in more sophisticated *in vivo* models. Our recent investigations using both wild type and transgenic zebrafish harboring rhabdomyosarcoma tumors have yielded very promising phototherapeutic outcomes, with detailed results to be published in the near future.

2.- Direct comparisons with other state-of-the-art photosensitizers (e.g., in terms of efficiency, cost of synthesis, or stability under physiological conditions) are underexplored and must be reported in the introduction.

- We thank the Reviewer for this valuable comment. In response, we have revised the introduction to include direct comparisons with other state-of-the-art type I photosensitizers. Owing to the concise letter format of our manuscript, we have provided a focused discussion that succinctly highlights these aspects without compromising the compact presentation required.

In this context, various design strategies have been employed to develop type I PSs.[35] For instance, isolated[36-38] and aggregation-induced emission (AIE)[39-41] luminogens—with either uncharged or positively charged forms—as well as supramolecular self-assembled dyes[42, 43] responsive to guest interactions[44] have been synthesized and characterized for type I photodynamic activity. However, some of these systems exhibit hybrid photodynamic mechanisms[36, 38], often combining type I and type II pathways or incorporating additional effects such as photothermal activation[37]. This mechanistic ambiguity complicates the interpretation of their photocytotoxicity, making it difficult to ascertain the exclusive contribution of the type I mechanism.

Moreover, several of these type I PSs possess extended chemical structures and high molecular weights[45, 46], which diverge from the drug-like properties essential for clinical translation. Another concern is that some of these agents exhibit half-maximal inhibitory concentration (IC_{50}) values under light irradiation in the 0.5–20 μM range, and some also show significant toxicity in the absence of light.[36, 39, 40] These factors collectively constrain their phototherapeutic indices and limit their overall clinical potential.

3.- As the interactions of TCI-NH with biomolecules, such as DNA and proteins, were demonstrated, more deep structural insights (e.g., through docking studies or crystallography) are missing. These would better elucidate binding modes and affinities. I suggest the authors include a paragraph related to this topic in the discussion.

- We thank the Reviewer for this valuable comment. In our study, our primary focus was on demonstrating that TCI-NH maintains its ability to generate superoxide species in both its free and biomolecule-bound states, which is critical for its photodynamic activity. While we agree that detailed structural insights would further clarify the binding interactions, our objective was not to perform an in-depth mechanistic analysis of the binding modes. Rather, we aimed to determine whether TCI-NH remains photodynamically active upon interaction with relevant biomolecules.

Based on previous studies with a closely related analogue (doi: 10.1093/nar/gkad365), we infer that TCI-NH likely binds to the *c-MYC* Pu22 G-quadruplex through external stacking interactions with the terminal guanine tetrads—a binding mode that is well documented for many ligands targeting this G4 structure. Similarly, with BSA, binding is expected to occur within the well-characterized hydrophobic pockets (commonly referred to as Sudlow sites I and II), as is typical for compounds exhibiting similar optical changes upon complexation. Given the significant experimental and computational efforts required for detailed molecular interactions studies—and the limitations inherent in preliminary in silico analyses—we chose not to pursue these approaches in the current work, as we believed that the data may be redundant, given the structural proximity of both compounds. Instead, we have now included a brief paragraph in the discussion that highlights these probable binding modes.

For example, Liu and co-workers reported that a 2′-hydroxychalcone derivative initially exhibited aggregation-caused quenching (ACQ) with a red-shifted emission band and underwent disassembly upon binding to human serum albumin (HSA).[58] Subsequently, the disassembled probe molecules were encapsulated within the hydrophobic cavity of HSA, which led to a marked fluorescence enhancement and a blue shift. The close agreement between these observations and our findings provided a strong basis for our mechanistic interpretation.

The reduced magnitude of the spectral shifts, compared to the BSA complex, suggests that although TCI-NH can engage in π-stacking interactions with the terminal guanine tetrads—as observed for its closely related analogue DBI[23] and numerous other ligands[59-62]—it remains partially exposed to the aqueous environment.

4.- Finally, the authors have only tested HeLa cells. Expanding the studies to other cancer cell lines or 3D tumour spheroids in a future paper could better demonstrate the generalizability of findings.

- We thank the Reviewer for this observation. We agree that evaluating TCI-NH in additional cancer cell lines and 3D tumor spheroid models would further substantiate the generalizability of our findings. In fact, our studies with a closely related analogue of TCI-NH demonstrated consistent photocytotoxic activity across different cancer cell types and tumor organoids (doi: 10.1093/nar/gkad365). Moreover, as mentioned in our response to Comment 1, we have recently performed *in vivo* studies using transgenic zebrafish harboring rhabdomyosarcoma tumors, which showed very promising phototherapeutic responses. We plan to expand these investigations to additional cancer models in future work.

Reviewer: 2

Recommendation: This paper is publishable subject to minor revisions noted. Further review is not needed.

Comments:

This manuscript reports about a very interesting and efficient PDT type I photosensitizer, which was tested on HeLa cells with very strong radical formation and cell death induction at relatively low concentrations and upon irradiation with blue light for relatively short time periods. Control experiments show that there the dark toxicity is negligible. Various characterization techniques (including extraterrestrial power radiation – EPR) were used to carefully analyze the cellular toxicity results. The PDT effect of the TCI-NH agent is impressive, the study is original and novel, and the results are interesting and impactful for the physical chemistry community. Unfortunately, the manuscript lacks clarity sometimes and many of my comments below may have been caused by misunderstanding of the experimental conditions and/or the results. I suggest that the authors clarify those points, which can probably be accomplished by minor but careful revision.

Comments:

Figure 2: Why were absorption, emission, and quantum yields measured in chloroform? And for cell imaging the TCINH was also in chloroform? It is not clear because only the concentrations are provided and a certain amount of DMSO is mentioned. Before the cell experiments, the TCI-NH should be analyzed in cell culture medium or other aqueous media.

- We thank the Reviewer for this comment. In our study, we measured the absorption, emission, fluorescence and singlet oxygen quantum yields of TCI-NH in $CHCl_3$ because this solvent—owing to its moderate polarity—effectively solubilizes the compound while minimizing aggregation effects. Moreover, direct measurements of singlet oxygen generation efficiency (using oxygen phosphorescence) are vastly favored in halogenated solvents, as the singlet oxygen lifetime and phosphorescence intensity is much higher, providing more accurate data. Under these conditions, the photophysical parameters accurately reflect the intrinsic behavior of isolated TCI-NH molecules and allow for direct comparison with parameters obtained for closely related analogues. For biological experiments, TCI-NH was initially dissolved in DMSO to prepare a stock solution. DMSO is widely used in such studies due to its excellent solvating power and its ability to dissolve both hydrophobic and hydrophilic compounds. Moreover, DMSO is miscible with aqueous media and, at the low concentrations employed (≤0.5% v/v), is well tolerated by HeLa cells without inducing cytotoxicity. This approach ensures that TCI-NH remains well dispersed when transferred into buffered aqueous solutions or cell culture media, thereby preserving its photophysical and photosensitizing properties under biologically relevant conditions.

  We hope this explanation clarifies our choice of solvents and underscores the robustness of our experimental approach.

The absorption and emission spectra, along with the photophysical properties of TCI-NH (excluding those involving biological templates or cellular studies, vide infra), were recorded in chloroform ($CHCl_3$)—a solvent of moderate polarity that effectively solubilizes the compound while minimizing aggregation effects (Figure 2).

For biological experiments, TCI-NH was dissolved in DMSO to prepare a stock solution—leveraging DMSO's ability to solubilize both hydrophobic and hydrophilic compounds, its miscibility with water and cell culture medium, and its low cytotoxicity in HeLa cells at concentrations ≤0.5% v/v.[16, 23, 24]

Supporting Information: A 5 mM stock solution of TCI-NH was prepared by dissolving the compound in DMSO. This solution was used for photosensitization studies in aqueous systems—including experiments both with and without biological matrices—as well as *in cellulo* assays. DMSO was selected not only to ensure high solubility but also to minimize aggregation. In subsequent biological assays, the DMSO concentration was carefully limited to 0.5% (v/v), a threshold that our cell culture studies have consistently shown to be non-cytotoxic.[3-5]

In fact, the following paragraphs mention that TCI-NH emits around 580 nm in aqueous. That means that the green signal in the imaging experiments in Figure 3 (495–560 nm) are probably not related to TCI-NH. On the other hand, interaction with proteins shifts the fluorescence back to the blue (527 nm) which means the green channel is maybe related to TCI-NH emission. I think spectroscopic analysis of TCI-NH in cell culture medium or better in a solution containing also cells, would be important.

- We thank the Reviewer for this comment, as It is important to clarify that in our cellular imaging experiments, unbound TCI-NH is removed during the washing steps, ensuring that the fluorescence signal primarily originates from the biomolecule-bound form of TCI-NH. As described in the manuscript, interaction with nucleic acids or proteins induces a blue shift in the emission of TCI-NH, placing it within the green channel (495–560 nm) used in Figure 3. Consequently, the observed signal accurately reflects the biomolecule-bound emission fingerprint of TCI-NH. Furthermore, each imaging channel was carefully calibrated to achieve the highest possible signal, and extending the fluorescence window for TCI-NH to longer wavelengths did not provide any additional benefit.

Cells were treated with 500 nM TCI-NH, incubated for 24 hours, and washed with phosphate-buffered saline (PBS) to remove unbound TCI-NH molecules.

The experiments concerning the non-production of singlet oxygen could be moved to the SI and the manuscript (if it remains in letter type) could simply mention that singlet oxygen production was negligible.

- We thank the Reviewer for this comment. All experiments related to singlet oxygen generation are provided in the Supporting Information.

ROS production experiments were again performed in chloroform?

- We thank the Reviewer for the comment. ROS production experiments investigating the photosensitization capacity of monodispersed TCI-NH were performed in organic solvents ($CHCl_3$ or DMSO). In contrast, ROS experiments involving biological matrices were conducted in aqueous solutions containing only negligible amounts of DMSO. This dual approach allowed us to assess both the inherent photosensitizing properties of TCI-NH and its performance in biologically relevant environments. Indeed, in both organic solvents and aqueous solutions, TCI-NH clearly demonstrated its ability to operate via a type I mechanism, with minimal singlet oxygen generation.

Figure 5B: How can the authors be sure that the green emission is CellROX because TCI-NH is also green (at least in the initial imaging experiments)? Maybe it is just TCI-NH emission and not ROS production?

- We thank the Reviewer for this comment. As stated in the manuscript, we employed CellROX™—a dye that is non-fluorescent in its native state but becomes strongly green fluorescent upon oxidation by ROS and subsequently localizes to the nucleus through DNA binding. In our experiments, the green fluorescence observed in Figure 5B (bottom panel) is localized exclusively in the nucleus, where TCI-NH does not emit. This distinct nuclear localization confirms that the green signal arises from oxidized CellROX™, thereby indicating ROS production.

Figure 6B: With such a strong phototoxicity, would one not expect that the irradiated cells in Figure 5B would look a little more damaged? They still look pretty healthy in those images. It would be great to specify the conditions in the figure captions, such that the results are comparable. Now one has to go back and forth between the manuscript text, the experimental part, and the figures to understand the different conditions (such as incubation times, solvents etc.). And, in fact, the cells do deform (Figure S10) but not in Figure 5B.

- We thank the Reviewer for this comment. In our experiments, cells were treated with TCI-NH and then either irradiated with light or maintained in the dark. Following 6 minutes of irradiation at 27 mW/cm^2 and subsequent incubation with CellROX™, the cells were immediately fixed with paraformaldehyde to arrest further cellular degradation. Additionally, in these experiments the entire well was irradiated, whereas in Figure S10 the light

  was confined to a specific spot for 12 minutes at 30 mW/cm^2. As a result, the cells in Figure 5B appear relatively healthy because the irradiation was not localized to a particular area and the fixation captured the cells at an early stage before severe phototoxic damage could manifest. In contrast, Figure S10 shows realtime cell deformation under continuous irradiation of the same area, revealing significantly more pronounced damage and clearly demonstrating photoinduced cell death.

However, under continuous irradiation of the same area, cells began to exhibit hallmarks of apoptotic cell death.[9, 16, 23, 24]

Figure S10: Why is DMSO (and not chloroform) used as control solution?

We thank the Reviewer for this comment. As mentioned above, in all experiments involving biological or cellular studies in aqueous solutions, we used a TCI-NH stock solution prepared in DMSO. DMSO is widely employed in such studies due to its excellent solvating power and its ability to dissolve both hydrophobic and hydrophilic compounds. Moreover, DMSO is fully miscible with aqueous media and, at the low concentrations used (≤0.5% v/v), is well tolerated by HeLa cells without inducing cytotoxicity.

Abbreviations EPR, TEMPO, DMPO, and TEMP wni (were not introduced). Why again solvent change (chloroform in the main experiments - I assume? – and DMSO in the controls)?

- We thank the Reviewer for this comment. We have now introduced the relevant abbreviations: EPR (electron paramagnetic resonance), TEMPO (2,2,6,6-tetramethylpiperidin-1-oxyl), DMPO (5,5-dimethyl-1-pyrroline Noxide), and TEMP (2,2,6,6-tetramethylpiperidine).

  Regarding the choice of solvents, the photophysical properties of TCI-NH were primarily investigated in $CHCl_3$ because its moderate polarity allows for complete solubilization of the compound while minimizing aggregation. This condition enables us to accurately assess the intrinsic behavior of isolated TCI-NH molecules and compare them with closely related analogues. However, it is well established that solvent polarity can influence the generation of specific ROS. Therefore, our EPR studies were performed using both $CHCl_3$ and DMSO. In $CHCl_3$—a solvent ideal for probing singlet oxygen production, as its natural lifetime is consistently enhanced in nonpolar and nonprotic solvents, in particular halogenated solvents—we observed negligible singlet oxygen generation by TCI-NH. In contrast, in DMSO, which is more polar and mimics the microenvironment of certain cellular compartments, we detected the formation of superoxide and photosensitizer-centered radicals, thereby underlining the specificity of TCI-NH for a type I mechanism.
